# Gene expression and agent-based modeling improve precision prognosis in breast cancer

**DOI:** 10.1038/s41598-025-01275-w

**Published:** 2025-05-16

**Authors:** Padmasri Sridharan, Mini Ghosh

**Affiliations:** Department of Mathematics, School of Advanced Sciences, Vellore Institute of Technology, Chennai, 600127 India

**Keywords:** Breast cancer, Gene expression profiling, Agent-based model, Survival predictions, Mathematical modeling, Machine learning, Cancer, Computational biology and bioinformatics, Biomarkers, Health care

## Abstract

Breast cancer survival is hard to predict because of the complex ways genes and cells interact. This study offers a new method to improve these predictions by combining gene expression profiling (GEP) with agent-based modeling (ABM). First, GEP will pinpoint genes that are important in breast cancer development. Then, a mathematical model will be built to show how these genes influence cell behavior. This data will be used in ABM to simulate tumor growth and treatment response. The ABM allows us to virtually test different treatments and see how they might affect patient survival. Finally, the model’s accuracy will be checked against real patient data and compared to other models. By combining the strengths of GEP and ABM, this research could significantly improve breast cancer survival prediction. ABM’s ability to analyze interactions mathematically could pave the way for more personalized and effective treatments.

## Introduction

Millions of women worldwide grapple with breast cancer, a complex and persistent challenge. Even with treatment advancements, the need for personalized therapies and accurate survival predictions remains. This complexity arises from breast cancer’s inherent unpredictability—how it progresses and how patients respond to treatment^1^. Traditional methods rely on factors like tumor size and hormone receptor status, which offer valuable information but fail to capture the intricate interplay between genes and cellular mechanisms that drive tumor behavior^2^.

This study proposes a groundbreaking approach to improve breast cancer survival prediction. It integrates gene expression profiling (GEP) and agent-based modeling (ABM) to unlock the secrets within cancer cells. GEP analyzes the molecular makeup of cancer by measuring the activity of hundreds of genes simultaneously^3^. This reveals gene signatures linked to specific breast cancer subtypes, disease progression, and treatment response. However, traditional GEP analysis often overlooks the dynamic interactions within the tumor microenvironment. Here’s where ABM comes in. It mimics the behavior of individual cells within a tumor and their interaction with surrounding tissues^4^. By incorporating mathematical models of biological processes, ABM can capture emergent features of a tumor, such as growth, invasion, and response to treatment.

This research leverages the strengths of both GEP and ABM through a unified mathematical framework. We use GEP data to identify genes with altered expressions linked to poor patient prognosis^5^. Statistical techniques like t-testing, fold-change analysis, and survival analysis models help achieve this. These findings are then translated into the ABM framework, requiring the definition of agent properties (like gene expression levels) and interaction rules based on the models. To ensure the simulated tumor behavior reflects real-world observations, we incorporate clinical data from breast cancer patients. We then calibrate the ABM by adjusting specific parameters through sensitivity analysis and parameter estimation techniques. Finally, we evaluate the model’s predictive ability using clinical datasets. Measures like the concordance index (C-index) and the area under the receiver operating characteristic curve (AUC) assess the model’s accuracy in predicting patient survival.

This research delves deeper into the following sections: Unveiling the Findings of the Integrated Approach, Interpreting the Outcomes and their Significance, Culminating the Journey: Key Research Findings, Unveiling the Fundamentals of GEP and ABM, Decoding the Genes for the ABM Model, Building the Unified Framework for Survival Prediction, Assembling the Model’s Data Arsenal.

## Background of the study

### Gene expression profiling (GEP)

Gene expression profiling (GEP) is a potent means in molecular biology enabling us to analyze gene activity in an assortment of cells or tissues^6^. The quantity of messenger RNA (mRNA) molecules that each gene releases must be tallied to figure out the activity level of a gene. Two widely employed techniques for grading gene expression are microarrays and RNA sequencing.

#### Microarrays

A surface with millimeter-sized dots that equate to target genes’ complementary DNA sequences; the mathematical model is based on foundational principles of chemical kinetics and systems biology^7,8^. In the dynamics of molecule $\:\left(B\right)$ that can bind to another molecule $\:\left(T\right)$ to form a complex $\:\left(BT\right)$, $\:B\left(t\right)$ represents the concentration of unbound molecules at time $\:t$. Similarly, $\:{k}_{h}\:$be the rate constant for the binding reaction $\:\left(B+T\to\:BT\right)$, $\:\left[T\right]\:$be the total concentration of molecule $\:T\:$(bound and unbound) where it is a constant here, $\:{k}_{d}\:$be the rate constant for the dissociation reaction $\:\left(BT\to\:B+T\right)$, $\:{B}_{max}\:$represents the maximum concentration of bound $\:B$ molecules (where all $\:B$ molecules are bound to $\:T$).

To express the concentration of unbound $\:B$ molecule $\:\left(B\left(t\right)\right)$ changes over time, it is represented by $\:\frac{dB\left(t\right)}{dt}$, which is the rate of change of $\:B$ with respect to time. There are two main processes affecting the concentration of unbound $\:B$, they are.

- *Binding* $\:B$ molecules can bind to $\:T$ molecules to form complexes $\:\left(BT\right)$. This process consumes unbound $\:B$ molecules and therefore has a negative impact on $\:\frac{dB\left(t\right)}{dt}$. The rate of binding is typically proportional to the concentration of both unbound molecule $\:\left(B\right)$ and the total concentration of $\:\left[T\right]$. By the law of mass action:
  $$\:{Rate}_{binding}={k}_{h}*B\left(t\right)*\left[T\right].$$
- *Dissociation* Bound $\:B$ molecules $\:\left(BT\right)$ can dissociate back into unbound $\:B$ and $\:T$. This process releases unbound $\:B$ molecules and therefore has a positive impact on $\:\frac{dB\left(t\right)}{dt}$. The rate of dissociation is typically proportional to the concentration of bound $\:B$ molecules $\:\left(BT\right)$
  $$\:{Rate}_{dissociation}={k}_{d}*BT\:.$$

And similarly,$\:{B}_{max}=B\left(t\right)+BT\:$(all molecules are either unbound or bound), substituting$\:\:BT\:$from the above to the alienation rate, then it becomes.

$$\:{Rate}_{dissociation}={k}_{d}*\left({B}_{max}-B\left(t\right)\right).$$

Now the overall rate of change of unbound $\:B$ $\:\left(\frac{dB\left(t\right)}{dt}\right)$ is the difference between the binding rate (negative) and the dissociation rate (positive):

1 $$\:{~~~~~~~~~~~~~~~~~~\hspace{7cm}\frac{dB\left(t\right)}{dt}=-\left({k}_{h}*B\left(t\right)*\left[T\right]\right)+{k}_{d}*\left({B}_{max}-B\left(t\right)\right)} \hspace{8.5cm} $$

#### RNA sequencing (RNA-seq)

With the use of this methodology, which comprehends individual RNA molecules, gene expression may be evaluated more quantitatively. The mathematical model is based on established principles of gene expression dynamics and reaction kinetics^9,10^. In a simple reaction system where a reactant $\:\left(R\right)$ converts into a product $\:\left(M\right)$, $\:R\left(t\right)$ represents the concentration of reactant molecules at time $\:t$ and $\:M\left(t\right)$ represents the concentration of product molecules at time $\:t$. $\:{k}_{t}$ is the rate constant for the reaction $\:(R\to\:M)$ where it is a constant rate of reactant disappearance and $\:{k}_{{d}_{r}}$ is the rate constant for the disappearance of reactant molecules, $\:{k}_{{d}_{m}}$ is the rate constant for the disappearance of product molecules. To define the rate of changes, $\:\frac{dR\left(t\right)}{dt}$ represents the rate of change of reactant concentration with respect to time and $\:\frac{dM\left(t\right)}{dt}$ represents the rate of change of product concentration with respect to time. There are two main processes affecting the concentration of reactant molecules $\:\left(R\right)$:

- *Disappearance of reactant* $\:R$ molecules dissolve due to the reaction that converts them into $\:M$. The rate of disappearance of $\:R$ is typically proportional to the concentration of $\:R$ itself. It is represented using the law of mass action, the negative sign indicates a decrease in reactant concentration.
  $$\:\frac{dR\left(t\right)}{dt}={-k}_{{d}_{r}}*R\left(t\right).$$
- *Appearance of product* $\:\:M$molecules can disappear due to various factors (degradation, consumption in other reactions). Since each disappearing reactant molecule leads to the creation of one product molecule, the rate of advent of $\:M$, $\:\frac{dM\left(t\right)}{dt}$ is often considered to be equal to the rate of disappearance of $\:R,\:\left({k}_{{d}_{r}}*R\left(t\right)\right)$. However, here we explicitly include a separate rate constant $\:\left({k}_{{d}_{m}}\right)$ for product disappearance.
  $$\:\frac{dM\left(t\right)}{dt}={k}_{{d}_{r}}*R\left(t\right)-{k}_{{d}_{m}}*M\left(t\right).$$

Considering the overall reaction rate $\:\left({k}_{t}\right)$ for reactant disappearance. This rate is often assumed to be constant and independent of the concentration of reactants (especially at the beginning when there is an abundance of reactants).

$$\:\frac{dR\left(t\right)}{dt}=-{k}_{t}*R\left(t\right)+{k}_{{d}_{r}}*R\left(t\right).$$

In some cases, depending on the specific reaction system, the disappearance rate of the reactant $\:\left({k}_{{d}_{r}}\right)$ might be directly related to the overall reaction rate constant $\:\left({k}_{t}\right)$. If a constant amount of reaction is continuously introduced into the system (maintaining a relatively steady concentration), then $\:{k}_{{d}_{r}}$ and $\:{k}_{t}$ can be interchangeable. Then the above two equations become:

2 $$\:\frac{dR\left(t\right)}{dt}={k}_{t}*R\left(t\right)-{k}_{{d}_{r}}*R\left(t\right)$$

3 $$\:\frac{dM\left(t\right)}{dt}={k}_{{d}_{r}}*R\left(t\right)-{k}_{{d}_{m}}*M\left(t\right)$$

By comparing the gene expression patterns of the tumor samples with the patterns of healthy controls, Differential Expression Analysis (DEA) and Machine Learning (ML) serve to gain insight into gene expression data from tumor samples. Examine whether there are any significant transcriptional differences (up- or down-regulated) amongst the genes.

### Agent-based modeling (ABM)

Agent-based modelling (ABM) is a computational mathematical modelling procedure that attempts to represent the actions and interactions of individual entities inside a system. For the context of the study of breast cancer, agents have been tapped to represent cancer cells, and the environment often reflects the tumor microenvironment. Deciphering the interactions between agents and their environment is key to understanding the dynamics of breast malignant cell behavior and tumor growth.

#### Agents

These depict distinct organisms possessing specific properties (such as cell type, size, and gene expression profile) and actions (like migration and proliferation). A drug for breast cancer cells that have, State variables include $\:X$ (Cell Size) and $\:Y$ (Level of Gene Expression).

#### Update rules

This specifies how the state of the agent varies over time.

4 $$\:\frac{dX}{dt}=f\left(Y,Nutrients\right)-d\left(death\:rate\right)$$

5 $$\:\frac{dY}{dt}=g\left(X,Signals\right)-h\left(degradation\:rate\right)$$

Eq. (4) describes cell size change based on gene expression $\:\left(Y\right)$ influencing growth rate $\:\left(f\right)$ and nutrient availability. A death rate $\:\left(d\right)$ is subtracted to account for cell death and Eq. (5) describes gene expression change based on cell size $\:\left(X\right)$ and external signals received (e.g., from neighboring cells). Gene expression degrades at a rate $\:\left(h\right)$.

#### Environment

This is an embodiment of the space in which agents remain and act. It comprises multiple agents (like blood vessels and immune cells) plus physical attributes (such as dietary availability and spatial organization). Two instances of how the environment may impact an agent’s behavior are nutritional gradient and interactions with other agents.

#### Interactions

Agents interact with the environment and with their fellow agents according to established norms. Certain responses (like immune cells annihilating cancer cells) and agent states (as with a breast malignant cell making growth factors that impact adjacent cells) can be driven by these interactions.

In breast cancer modelling, agents are permitted to represent various cell types, comprising endothelial, immune, and cancer cells. The tumor microenvironment is reflected in the environment of the tumor^11,12^. Cells are positioned on a spatial grid in discrete locations. Nutrient diffusion alters cell growth as nutrients move over the grid and links between cells, cancer cells can thwart immune responses, compete for nutrients, and produce signaling molecules.

## Methodology

### Schematic representation

**Fig. 1:**
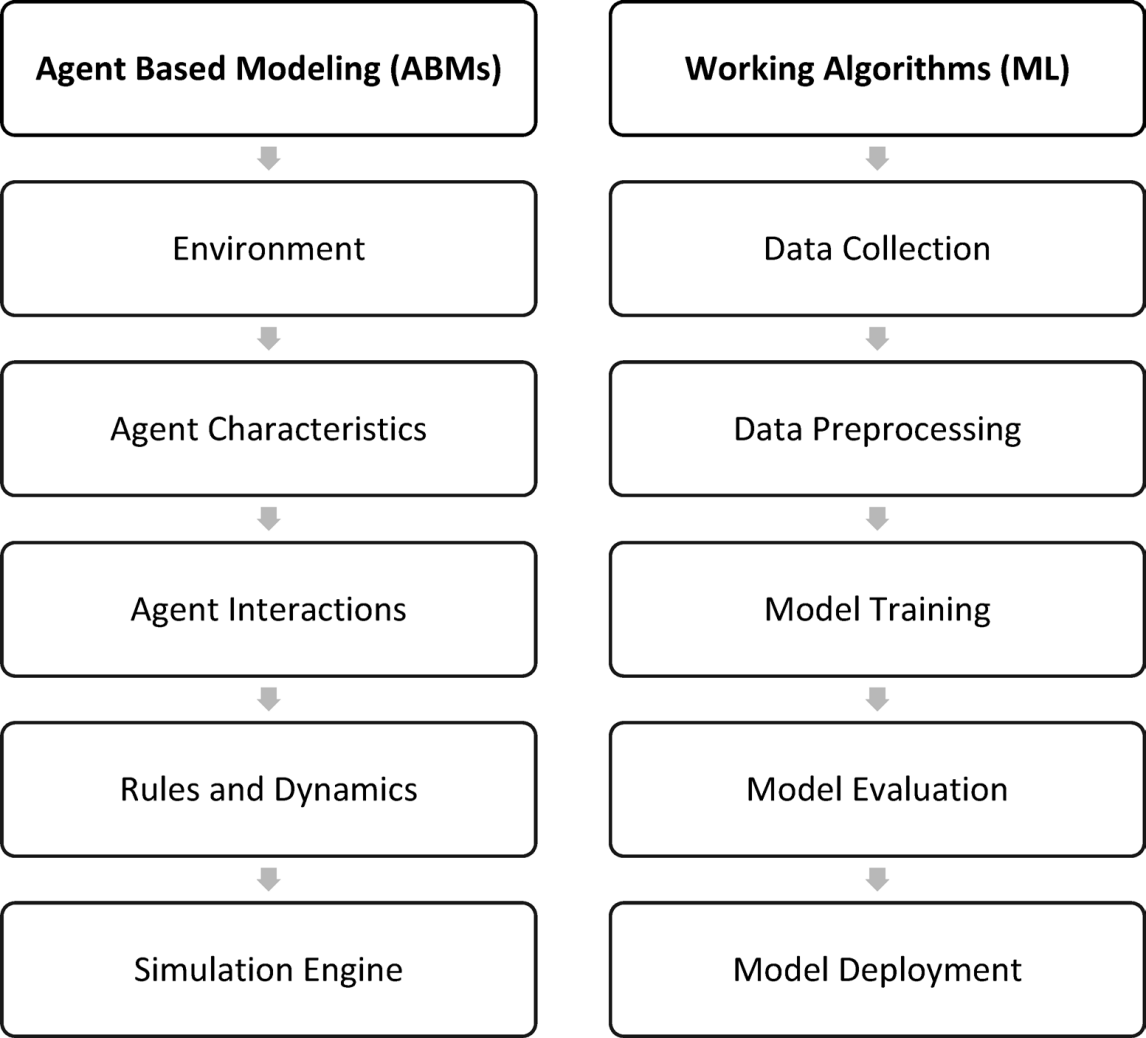
ABMs and ML: a symbiotic union.

Insights from real-world data are rendered by computerized learning (ML), as represented in Fig. 1. As ABM fosters a dynamic simulation of a patient’s breast cancer growth utilizing the insights from algorithmic learning, it aids in identifying the most crucial components for survival prediction and the possible linkages between them. Testing the outcomes of different therapies on patient survival and assessing “what-if” scenarios are rendered conceivable by this.

Benefits of the conjugation include the ability to generate more accurate models for predicting breast cancer survival by fusing the simulation drives of ABM with the pattern recognition of ML^13,14^. Tailored forecasts and therapy plan that take note of unique variations are made feasible by ABM, which makes it more facile to perceive the subtle relationships between variables impacting patient survival and the trajectory of breast cancer.

### Data sources

The expression levels of numerous genes in the tumor samples are depicted by gene expression data, which is primarily gathered via RNA-sequencing or microarray examinations. A matrix is employed in this format. Patient samples are expressed by columns, genes by rows, and the expression level of each gene $\:\left({M}_{ij}\right)$ in patient sample $\:j\:$is shown by each entry.

$$\:{M}_{ij}=\left(\begin{array}{ccccc}{m}_{11}&\:{m}_{12}&\:{m}_{13}&\:\dots\:\dots\:\dots\:\dots\:&\:{m}_{1j}\\\:{m}_{21}&\:{m}_{22}&\:{m}_{23}&\:\dots\:\dots\:\dots\:\dots\:&\:{m}_{2j}\\\:{m}_{31}&\:{m}_{32}&\:{m}_{33}&\:\dots\:\dots\:\dots\:\dots\:&\:{m}_{3j}\\\::&\::&\::&\:\dots\:\dots\:\dots\:\dots\:&\::\\\::&\::&\::&\:\dots\:\dots\:\dots\:\dots\:&\::\\\::&\::&\::&\:\dots\:\dots\:\dots\:\dots\:&\::\\\:{m}_{i1}&\:{m}_{i2}&\:{m}_{i3}&\:\dots\:\dots\:\dots\:\dots\:&\:{m}_{ij}\end{array}\right)$$

The process of gene expression is intricate and incorporates multiple state regulatory mechanisms. Nevertheless, it can be described mathematically, with the following equation defining the rate of change in gene expression as the system likely represents the dynamic molecule denoted by $\:M$. $\:{M}_{ij}\left(t\right)$ represents the concentration of this molecule $\:M$ at location $\:(i,j)$.

This suggests a spatially distributed system, possibly on a grid or lattice. $\:{k}_{{trans}_{i\left(t\right)}}$ represents the rate of evolution or creation of molecule $\:M$ at location $\:(i,j)$ at time $\:t$, this rate might be time-dependent and $\:{k}_{{deg}_{i}}$ represents the degradation rate constant of molecule $\:M$, which is assumed to be constant across space (independent of location). There are two main processes affecting the concentration of $\:{M}_{ij}\left(t\right)$.

#### Transition/creation rate

$\:M$ Molecules can appear at location $\:(i,j)$ due to various processes like transport from neighboring locations or local production. This rate might depend on the local concentration of $\:M$ or other factors and might vary over time. This process has a positive impact on $\:\frac{{dM}_{ij}\left(t\right)}{dt}$ it is represented as a general function $\:{k}_{tran{s}_{i\left(t\right)}}$.

#### Degradation rate

$\:M$ molecules can disappear from location $\:(i,j)$ due to degradation or conversion into other molecules. The degradation process is often assumed to be proportional to the current concentration of $\:M$ at that location. This process has a negative impact on $\:\frac{{dM}_{ij}\left(t\right)}{dt}$.

Therefore, the degradation rate is represented by, $\:{k}_{{deg}_{i}}*{M}_{ij}\left(t\right)$. The overall rate of change of $\:{M}_{ij}\left(t\right)$ is the difference between the rate of transition/creation and the rate of degradation:

6 $$\:\frac{d{M}_{ij}}{dt}={k}_{{trans}_{i\left(t\right)}}-{k}_{{deg}_{i}}*{M}_{ij}\left(t\right)$$

The above Eq. (6) describes the concentration of molecule$\:\:M\:$at a specific location$\:\:\left(i,j\right)$ changes over time based on the rates of local creation and degradation. It provides information about various factors that can potentially influence gene expression and tumor development.

### Gene selection

Selecting the class of genes that are vital for survival outcome prediction is the intent^15,16^. Genes constitute elements in this logical sense. The methods we employ are as follows:

#### Differential expression analysis

Analyze if gene expression shifts between statistically relevant patient combinations, such as those with a good or poor prognosis. This is accomplished by using statistical tests such as ANOVA and t-tests. The approach involves mathematical phases such as comparing the distribution of $\:\left({M}_{ij}\right)$ across numerous patient groups and locating genes where the distribution fluctuates significantly.

#### Machine learning techniques

By training algorithms on the entirety of the gene expression dataset and survival data, a subset of genes that are exceptionally accurate of survival outcomes gets found. It entails tactics like Lasso Random Forests. By cultivating extensive models and analyzing the relationships between gene expression patterns $\:\left({M}_{ij}\right)$ and survival outcomes, an array of major genes have been found. A gene’s role in the onset of breast cancer or its response to therapy may be ascertained by prioritizing it for more study. The selection process relies on the body of available research and knowledge of relevant signaling pathways.

The decision-making process is based on stated biological data about genes$\:\:\left({G}_{i}\right)$ and their known duties in cancer biology, as indicated by $\:\left({R}_{i}\right)$. Genes with widely researched functions $\:\left({R}_{i}\right)$ tied to drug resistance, metastasis, or tumor growth are chosen. We can narrow down a subset of genes with biological and statistical significance that ought to be featured in the ABM by combining them.

### Agent-based model (ABM)

An agent-based model (ABM) for a study of breast cancer dynamics is built by first developing an entity of agents, then defining their behavior rules, and then calibrating and validating the model adopting experimental data.

#### Design of agents

- *Breast cancer cells* Acts as primary agents, with attributes like, position $\:(X,Y)$ on a spatial grid representing the tumor microenvironment, size $\:\left(S\right)$, and gene expression profile (represented by a vector of expression levels, $\:E=\left\{{E}_{1},{E}_{2},{E}_{3},\dots\:,{E}_{n}\right\}$, for $\:n\:$genes).
- *Other components* There is additionally proposed of immune cells with criteria like activation state, killing state, and zone of residency, such as cytotoxic T cells, as added agents. Endothelial cells qualify due to the reflected aspects of artery walls, such as site and rate of nutrient transport.

#### Defining agent behavior rules

The rules control how an agent updates its state, including dimensions, positioning, and gene expression—in response to interactions with other agents and the environment. The depiction of the mathematical model is as follows:

- *Breast cancer cell update rules* The system represents the growth of a tumor or a specific cell population within a tumor. $\:S\left(t\right)$ represents the size or volume of the tumor (or the number of cells in the population) at time $\:t$.$\:{\:E}_{growth}\:$represents a factor influencing the growth rate of the tumor/cell population, this could be a variable like nutrient availability, growth factors or a combination of these, $\:N\:$represents another factor affecting growth, possibly the current population size (cell number) or a measure of the tumor microenvironment and $\:d$ represents the rate of cell death within the tumor/cell population, assumed to be constant. There are two main processes affecting the tumor size $\:\left(S\right)$.
- *Growth and death rates* The tumor/cell population grows due to cell proliferation and expansion. The growth rate is typically influenced by various factors. The number of tumor cells at time $\:t$ is $\:N\left(t\right)$ based on logistic growth.
  7 $$\:\frac{dN}{dt}=r*N\left(t\right)*\left(1-\:\frac{N\left(t\right)}{K}\right)$$
  where $\:r$ is the proliferation rate constant and $\:K$ is the carrying capacity of the tumor microenvironment. The Eq. (7) describes the tumor size changes over time based on a function representing the growth rate (influenced by factors like nutrient availability and population size) and a constant death rate.

The equation for the Gene Expression $\:\left(E\right)$ depicts how gene expression changes based on internal factors (cell size) and external stimuli (signals), with a constant degradation term. $\:{E}_{i}\left(t\right)$ represents the concentration or level of the cellular component at time $\:t$, $\:S$ represents the size or volume of the surrounding tumor or cell population (potentially influencing the dynamics of $\:{E}_{i}$).

Due to the $\:Signals$ which represents various external factors (e.g., growth factors, signaling molecules) that can affect the level of$\:\:{E}_{i}$, $\:{g}_{i}\left(S,Signals\right)$ represents a function capturing the overall rate of change (production or activation) of$\:{\:E}_{i}$. This rate might depend on the tumor size $\:\left(S\right)$ and the external signals and $\:{h}_{i}\:$represents a constant rate of degradation or removal of $\:{E}_{i}$. The processes affecting the level of $\:{E}_{i}\left(t\right)$:

I. *Production/activation rate* $\:{E}_{i}\:$can be produced or activated due to various factors like cell signaling or internal processes within the cell. It is represented by a function$\:\:{g}_{i}\left(S,signals\right),\:\text{t}$his function captures how the production/activation rate depends on the surrounding tumor size $\:\left(S\right)$ and the presence of external signals and has a positive impact on $\:\frac{d{E}_{i}}{dt}$.
II. *Degradation/removal rate* $\:{E}_{i}\:$can be degraded, removed, or deactivated through various mechanisms. The degradation process is often assumed to be proportional to the current level of $\:{E}_{i}$. Therefore, the degradation rate can be represented by a constant rate term $\:{h}_{i}$ and has a negative impact on $\:\frac{d{E}_{i}}{dt}$. The overall rate of change of $\:{E}_{i}\left(t\right)$ is the difference between the production/activation rate and the degradation/removal rate,
  8 $$\:\frac{d{E}_{i}}{dt}=Signals*{E}_{i}\left(t\right)*\left(1-\:\frac{{E}_{i}\left(t\right)}{{h}_{i}}\right)$$

The Eq. (8) describes the level of the cellular component $\:{E}_{i}$ changes over time based on functions representing its production/activation (influenced by factors like tumor size and gestures) and a constant degradation rate.

c. *Immune cell update rules* It involves movement towards cancer cells, activation based on encountering cancer cells, and a killing rate impacting cancer cell size.

d. *Environment update rules* It involves nutrient diffusion based on predefined rules and consumes nutrients as cancer cells grow.

#### Calibration/validation

- *Calibration* To ensure the model’s behavior aligns with biological observations, we employed a multi-step calibration process. First, we used sensitivity analysis to identify the most influential parameters, such as growth rate constants $\:\left({k}_{growth}\right)$ and gene-linked factors $\:\left({E}_{i}\right)$. These parameters were then tuned using a combination of gradient-based optimization and Monte Carlo simulations to minimize the error between simulated and experimental data on tumor size development and cell proliferation rates. Specifically, we used the Nelder-Mead simplex method for gradient-free optimization and Markov Chain Monte Carlo (MCMC) sampling to explore the parameter space. This approach ensures that the model parameters are robust and biologically plausible. The calibrated parameters were then used to update the agent behavior rules (Eqs. 7 & 8).
- *Validation* To validate the model, we compare its predictions with clinical observations and independent experimental data. We employed a 10-fold cross-validation approach, where the dataset was split into training and testing subsets. The model was trained on 90% of the data and tested on the remaining 10%, with this process repeated 10 times to ensure robustness. We evaluated the model’s performance using metrics such as the Concordance Index (C-Index) and the Area Under the Receiver Operating Characteristic Curve (AUC-ROC). Additionally, we compared the simulated tumor growth curves with clinical tumor development data from the METABRIC, TCGA-BRCA, and GSE96058 datasets. The validation results showed strong agreement between the model’s predictions and real-world observations, confirming the model’s accuracy and reliability.

To validate the model’s generalizability, we independently applied it to two external cohorts: TCGA-BRCA (*N* = 1098) and GSE96058 (*N* = 3273). Gene expression data were harmonized using ComBat batch correction, and clinical variables (e.g., tumor stage, lymph node status) were mapped to METABRIC’s schema. Model performance was evaluated via 10-fold cross-validation, with metrics reported for each dataset (Table 2).

## Integration framework

**Fig. 2:**
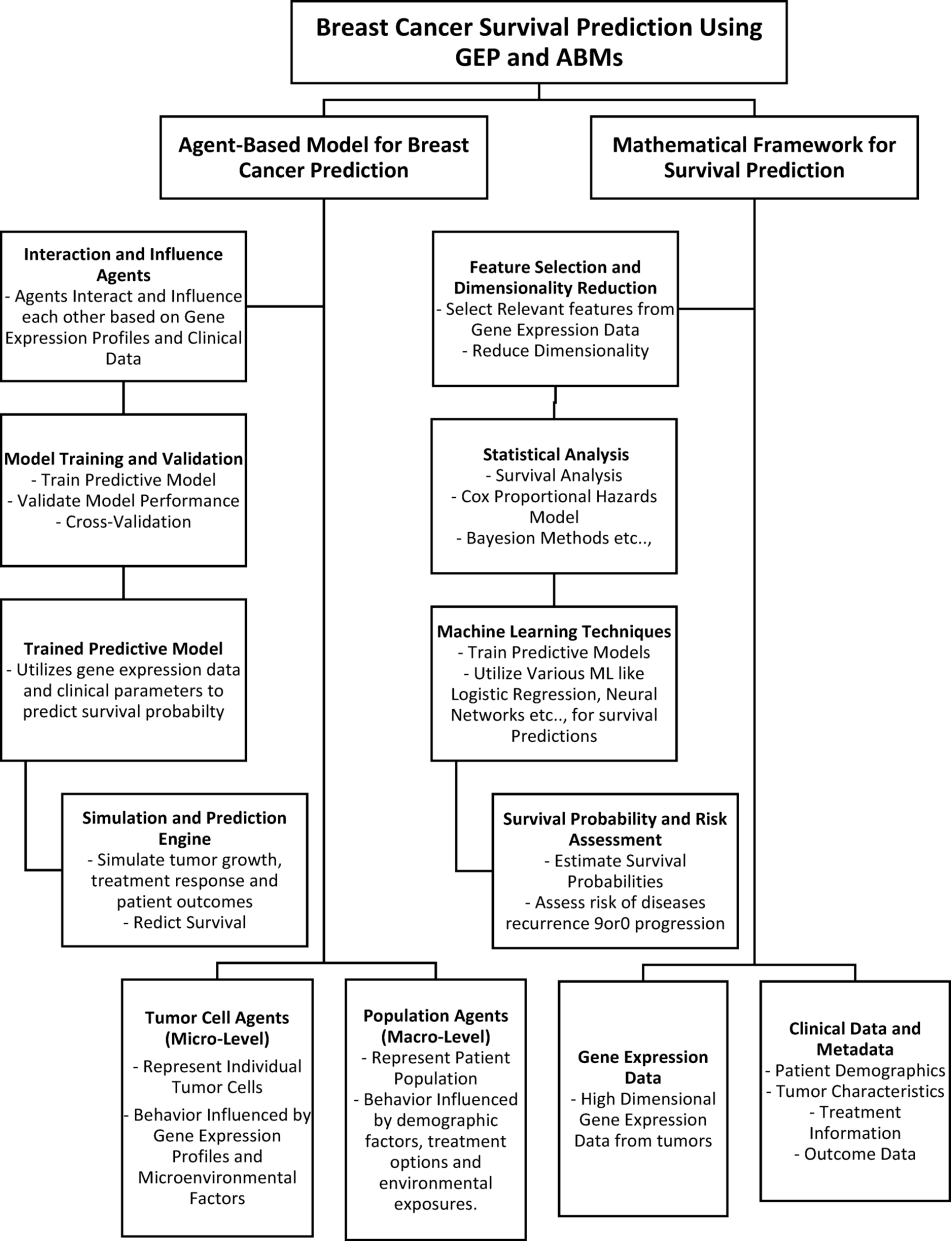
Breast cancer survival prediction using gene expression profiling.

In the context of predicting breast cancer survival using gene expression and agent-based modelling, the contents of Fig. 2 provide an adequate diagrammatic depiction that covers the data-driven modelling unit. These components involve tapping into clinical information and gene expression data to regulate the behavior and interactions of the agents in the agent-based model. It also involves fitting the mathematical framework’s parameter estimates and model validation to multiple sources. Based on real-life data, data-driven modelling ensures that the agent-based model and mathematical framework appropriately portray the complexity of patient outcomes and dynamics concerning breast cancer^17,18^. To integrate gene expression data with the Agent-based Model (ABM), we explore how the expression levels modify the traits and actions of the agents. This often entails a multi-step process based on mathematical equations to shed light on the dynamics of gene expression and its impact on the ABM.

### Linking gene expression to agent properties and behaviors

Gene expression data ($\:E=\{{E}_{1},{E}_{2},\dots\:\dots\:\dots\:\dots\:,{E}_{n}\}$) informs the initial state and update rules governing breast cancer cell agents within ABM. Here is how the connection is made to be established.

#### Initial state

The initial expression profile $\:\left(E\right)$ for cancer cell agents is established by the levels of gene expression in a patient sample. This renders the model unique to the tumor biology that exists in the patient; an initial requirement that a specific gene $\:\left(i\right)$ is expressed as,

9 $$\:{M}_{i}\left(t=0\right)={E}_{i}\left(patient\:data\right)$$

which translates to the initial expression level $\:{M}_{i}\:$for gene $\:i$ in the ABM being set to the corresponding value $\:{E}_{i}\:$obtained from the patient’s gene expression data.

#### Update rules

The update rules for cancer cell size $\:\left(S\right)$ and gene expression $\:\left(E\right)$ are influenced by the gene expression data, as from Eq. (7), $\:{E}_{growth}$ can be weighted sum of expression levels for genes involved in cell cycle progression $\:\left({G}_{growth}=\left\{{G}_{1},{G}_{2},\dots\:\dots\:\dots\:\dots\:,{G}_{m}\right\}\right)$. Each gene’s contribution can be weighted by a coefficient $\:{W}_{j}\:$reflecting its relative importance. Mathematically it is defined as it captures the influence of multiple genes $\:{E}_{i}$ on growth rate such as:

10 $$\:{E}_{growth}=\sum\:\left({W}_{j}*{E}_{j}\right)for\:j\in\:\left(\text{1,2},\dots\:\dots\:\dots\:\dots\:,m\right)gene\:in\:{G}_{growth}$$

Similarly, from Eq. (8) signals can be influenced by the presence of other agents like immune cells and their activation state, which might be linked to specific gene expression patterns observed in the patient data. For instance, the presence of activated immune cells $\:\left(A\right)$ could downregulate a growth-related gene $\:\left(i\right)$ through a specific signaling pathway, where $\:A\:$represents the concentration of another molecule (Activator) that can interact with $\:{E}_{i}$ and $\:k$ represents the rate constant for the interaction between $\:{E}_{i}$ and $\:A$. There are three main processes affecting the level of $\:{E}_{i}\left(t\right)$:

- *Production/activation rate* This rate is captured by the function $\:{g}_{i}\left(S,Signals\right)$ as explained previously in the Eq. (8) formulation.
- *Degradation/removal rate* This rate of process is also captured by $\:{h}_{i}$ which is explained previously in the Eq. (8) form.
- *Interaction rate* $\:{E}_{i}\:$interact with molecule $\:A$. The nature of the interaction depends on the specific system being modeled. Here, we represent the rate of change due to this interaction with a term $\:\pm\:k*A$, where the sign depends on whether $\:A$ consumes or produces $\:{E}_{i}$.

I. *Positive* $\:k$ if $\:A$ consumes $\:{E}_{i}$ during the interaction, the rate of change is negative $\:\left(-k*A\right)$, signifying a decrease in $\:{E}_{i}$ due to this interaction.
II. *Negative* $\:k$ if $\:A$ promotes the production of $\:{E}_{i}$ through the interaction, the rate of change includes a positive term $\:\left(+k*A\right)$, signifying an increase in $\:{E}_{i}$.

The overall rate of change of $\:{E}_{i}\left(t\right)$ is the sum of the rates due to production/activation, degradation/removal, and interaction with $\:A$:

11 $$\:\frac{d{E}_{i}}{dt}={g}_{i}\left(S,Signals\right)-{h}_{i}-k*A$$

The above Eq. (11) describes the level of the cellular component $\:{E}_{i}$ changes over time based on functions representing its production/activation (influenced by factors like tumor size and signals), a constant degradation rate, and the interaction with another molecule $\:\left(A\right)$ represented by a rate constant $\:\left(k\right)$ and its concentration.

#### Initialization and parameterization (patient-specific data)

Patient-specific data like tumor size/volume $\:\left({V}_{o}\right)$ and stage $\:\left({S}_{stage}\right)$ can be used to initialize the number $\:\left({N}_{o}\right)$ and distribution of cancer cell agents within the ABM environment. A relationship between initial tumor size/volume $\:\left({V}_{o}\right)$ and the number of initial cancer cell agents $\:\left({N}_{o}\right)$ as:

12 $$\:{N}_{o}=\frac{{V}_{o}}{average\:cell\:size}\:$$

This establishes a link between patient data $\:\left({V}_{o}\right)$ and the model initialization $\:\left({N}_{o}\right)$ by the concept of proportionality.

### Simulating tumor growth and survival with ABM

To figure out how to forecast survival outcomes associated with breast cancer research, it examines the simulation process of the agent-based model (ABM). Although ABMs execute discreetly, we will be recommending that they function as follows:

#### Running the ABM

The ABM iterates through discrete time steps $\:t=(\text{1,2},3,\dots\:)$ simulating the dynamic changes within the tumor microenvironment. Here is the proposition of the breakdown of the process with mathematical equations:

Firstly, the update rules govern how each agent (cancer cell, immune cell) modifies its state (i.e., position, size, gene expression) based on interactions with the environment and other agents in each time step, from Eq. (7) by capturing the rate of change and updating rule for cancer cell size $\:\left(S\right)$ it becomes a discrete update in the ABM as:

13 $$\:\varDelta\:S\left(t\right)=f\left({E}_{growth}\left(t-1\right),N\left(t-1\right)\right)-d\:$$

where $\:\varDelta\:S\left(t\right)\:$represents the change in the size or volume $\:\left(S\right)$ of a biological entity between time $\:t-1$ and time $\:t$. It basically captures the growth experienced by the entity during that time interval. $\:f\left({E}_{growth}\left(t-1\right),N\left(t-1\right)\right)$ it represents a function $\:\left(f\right)$ that calculate the growth experienced by the entity based on two factors at the previous time step $\:\left(t-1\right)$, $\:{E}_{growth}\left(t-1\right)\:$represents a value related to factors influencing growth, possibly the availability of resources or growth factors at the previous time step and $\:N\left(t-1\right)$ represents the size or volume $\:\left(N\right)$ of the entity at the previous time step. The growth function $\:\left(f\right)$ might depend on the current size of the entity $\:\left(N\right)$ as well and $\:d$ represents a constant rate of death or loss of volume within the entity. While the Eq. (13) related to an ODE model for growth, i.e.,

$$\:\frac{dS}{dt}=g\left(S,{E}_{growth}\right)-d$$

Here $\:\frac{dS}{dt}$ represents the rate of change of the size $\:\left(S\right)$ at any given time $\:\left(t\right)$ and $\:g\left(S,{E}_{growth}\right)$ represents a function capturing the growth rate, potentially depending on the current size $\:\left(S\right)$ and growth factors $\:\left({E}_{growth}\right).$ The $\:\varDelta\:S\left(t\right)$ equation is seen as an approximation of the change in size $\:\left(dS\right)$ over a specific time interval $\:\left(t\:to\:t-1\right)\:$using values from the previous time step and a constant death rate.

Secondly, tumor growth simulation over multiple time steps the ABM tracks the overall changes in the cancer cell population. This has been represented as:

14 $$\:N\left(t\right)=N\left(t-1\right)+\varDelta\:N\left(t\right)$$

In this model, $\:N\left(t\right)$ represents the number of cancer cells at time $\:t$. This quantity influences tumor progression and treatment response. The growth rate $\:N\left(t\right)$ follows a balance between proliferation and cell death.

Where $\:\varDelta\:N$ represents the net change in cell number due to growth, death or division which is like the concept of population growth models in ecology, where the current population size depends on the previous size and birth/death rates. By iterating this equation, the ABM simulates the growth of the tumor over time interval $\:\left(t\:to\:t-1\right)$.

Eventually, to evoke the impact of immunotherapy on the tumor microenvironment, we offer immune cell agents with elevated activity or proliferation rates. From the medium of update rule renditions or the addition of additional agents, the ABM can simulate the effects of various treatments on the dynamics of breast cancer progression. The agent that acts as a surrogate for the chemotherapeutic medication and interacts with cancer cells to either slow down or kill them may require tweaking the cell size *(S)* enhancing rule to cater for this interaction.

#### Extracting relevant metrics

Following an agreed-upon period, the simulation takes place, and pertinent metrics are retrieved to evaluate potential patient outcomes in the following way.

Time to Relapse, monitor the simulated tumor for virtual relapse events. This might be defined as exceeding a critical size threshold $\:\left({S}_{threshold}\right)$ or reaching a specific spatial location like mimicking metastasis. Mathematically, the relapse time $\:\left({T}_{relapse}\right)$ can be identified when the simulated tumor state of size reaches the relapse criterion,

15 $$\:{T}_{relapse}={min}\left(t:S\left(t\right)\ge\:{S}_{threshold}\right)$$

where $\:{min}\left(t:S\left(t\right)\ge\:{S}_{threshold}\right)$ defines the searches for the minimum value of time $\:\left(t\right)$ for which the condition $\:S\left(t\right)\ge\:{S}_{threshold}$ is satisfied and $\:S\left(t\right)$ represents a quantity like tumor size, number of infected cells that changes over time $\:\left(t\right)$ within the model. $\:{S}_{threshold}\:$represents a precise threshold value for the quantity $\:S\left(t\right)$ it represents a critical level of disease progression that signifies a relapse, used to assess treatment efficacy, or predict the time window for intervention in the model.

Overall Survival, simulate the duration of the virtual tumor until a predefined endpoint $\:\left({T}_{max}\right)$ where it reaches a specific size, exceeding a time limit, or experiencing a virtual death event due to treatment effects. Overall survival $\:\left(OS\right)$ container be calculated as the simulated time until the endpoint,

16 $$\:OS={T}_{max}$$

Population dynamics and rates of change are key to the ABM simulation methodology. The above-discussed equations and dynamics permit us to predict survival in a virtual tumor environment and explore potential therapy effects. By evaluating $\:OS$ values, we can assess the effects of various factors like treatment strategies and genetic mutations on overall survival or disease progression time. The equation aids in summarizing the findings of a model simulation related to disease progression or survival. The opted-for value of $\:{T}_{max}\:$provides a single metric for comparison across different simulations or treatment scenarios within the model.

## Description of the model

Breast cancer is one of the most prevalent kinds of cancer and costs a lot of cash to cure. One of the barriers to optimizing resources yet offering more individualized treatment is accurately predicting how different individuals will respond to therapy. Age, neoplasm histologic grade, tumor size, tumor stage, and the number of positive lymph nodes are the most significant factors that we take into perspective. Parallel studies yielded similar predictions for these top attributes. The logistic regression classifier yielded a mean AUC-ROC of 84.5% for a grouping $\:t\le\:7$. Overall, the logistic function time derivation technique employs linear approximation to predict the patient’s future likelihood of survival based on clinical information, genetic mutations, and expertise.

Forecasting accurately the duration of treatment is critical in clinical decision-making to optimize resources, modify prognosis, and configure therapeutic doses^19,20^. Even when an individual’s clinical features and illness stage are the same, genetic factors may dictate how well they tolerate treatment and their overall prognosis. Cancer begins with anomalies or mutations in the DNA. It may be carried out in several ways, notably DNA microarray. Despite numerous studies that have been conducted to predict the survival of individuals with breast cancer using clinical data, just a handful of studies employed data mining/machine learning strategies to predict outcomes using clinical variables.

We merged genetic and clinical data into the framework we designed to predict a breast cancer patient’s prognosis at a given period. We seek to respond to these two primary queries:

1. What are the most significant predictors of a patient’s prognosis in breast cancer?
2. What is the patient’s chance of survival over the next $\:t$ years, given their clinical and genetic characteristics?

### Integration approach

The ABM simulation was implemented using a discrete-time iterative framework in Python. Each agent (tumor cell) was initialized with gene expression values from patient data. The simulation tracked cell proliferation, apoptosis, and treatment effects over time. The model was validated against clinical datasets. The full implementation, including code and dataset preprocessing scripts, is available at our GitHub repository, as described in the Code availability section.

#### Performance evaluation

- *Concordance index (C-index)* This metric assesses how well the model ranks patients based on their predicted survival outcomes. A C-Index of 1 indicated perfect agreement, while 0.5 indicates random prediction, where a group of patients $\:\left(n\right)$ with known survival times $\:\left({T}_{actual}\right)\:$and model-predicted survival times$\:\:\left({T}_{predicted}\right)$. The C-index is calculated as:
  17 $$\:C-Index=\frac{1}{\left(n*\left(n-1\right)\right)*\sum\:\left(I\left({T}_{{predicted}_{i}}>{T}_{{predicted}_{j}}\right)*\left({T}_{{actual}_{i}}>{T}_{{actual}_{j}}\right)\right)}\:$$
  $\:I\left({T}_{{predicted}_{i}}>{T}_{{predicted}_{j}}\right)$ is an indicator function, it equals 1 if the predicted event time for observation $\:i\left({T}_{{predicted}_{i}}\right)$ is greater than the predicted event time for observation $\:j\left({T}_{{predicted}_{j}}\right)$, and 0 otherwise, and $\:\left({T}_{{actual}_{i}}>{T}_{{actual}_{j}}\right)$ term checks the actual order of events, it equals 1 if the actual event time for observation. $\:i\left({T}_{{actual}_{i}}\right)$ is greater than the actual event time for observation. $\:j\left({T}_{{actual}_{j}}\right)$, and 0 otherwise, where $\:i$ *and* $\:j$ iterate over all patient’s pairs, $\:I\left(.\right)$ is the indicator function (1 if true, 0 otherwise). It is for the concept of ranking and comparisons, assesses how well the model aligns with actual survival data.
- *Calibration curves* It exhibits the discrepancy between the model’s predicted survival probabilities and the actual observed survival rates at different moments. In this scenario, it is ideal for the observed and predicted probabilities to coincide.

#### Identifying key genes/pathways

We have identified significant genes or pathways controlling survival by examining the updated rules and their reliance on gene expression levels $\:\left(E\right)$ in the ABM. These genes include:

- *Sensitivity analysis* Executing the model with various factors corresponding to everyone’s gene expression level $\:\left({E}_{i}\right)$ and observing how it impacts expected survival outcomes (such as the time to relapse). Substantial variations in survival outcomes that are correlated with shifts in their expression level constitute potentially relevant genes.
- *Virtual knockouts* In the computational framework, toggle destined gene expressions $\:\left(set\:{E}_{i}\:to\:0\right)$ and investigate the impact on the growth and survival of a simulated tumor. Genes which greatly enhance survival outcomes after virtual deletion should be deemed potential therapeutic targets.

### Data description

The model implemented in the study by Pereira et al. (2016) included a dataset from the British Columbia Cancer Centre in Canada that was published in Nature Communications. The dataset includes 31 clinical factors (patient age, tumor stage, size, and type of breast cancer) paired with 331 genetic characteristics (z-scores based on genetic profiles, 175 genetic mutations, and mRNA). The 1980s are emphasized with instances. To enhance the generalizability of our findings,

The clinical and genomic features used in our model are summarized in Table 1, which includes patient demographics, tumor characteristics, and treatment-related variables. These features were preprocessed and integrated into the ABM framework to ensure patient-specific simulations.”

**Table 1:** Data description of the data to be used in the working model.

S. no	Features	Description
1	patient_id	Patient ID
2	ageatdiagnosis	Age of the patient at diagnosis time
3	typeofbreast_surgery	Breast cancer surgery type (1) MASTECTOMY (removal of all breast tissue from a breast) (2) BREAST CONSERVING (part of the breast that has cancer is removed)
4	cancer_type	Breast cancer types (1) Breast cancer (2) Breast sarcoma
5	detailed_cancer_type	Detailed Breast Cancer types (1) Breast Invasive Ductal Carcinoma (2) Breast Mixed Ductal and Lobular Carcinoma (3) Breast Invasive Lobular Carcinoma (4) Breast Invasive Mixed Mucinous Carcinoma (5) Metaplastic Breast Cancer
6	Cellularity	Cancer cellularity post chemotherapy (amount of tumor cells in the specimen and their arrangement into clusters)
7	chemotherapy	Whether or not the patient had chemotherapy as a treatment (Yes/No)
8	pam50 + claudin-low_subtype	Pam 50: is a tumor profiling test that helps show whether some estrogen receptor-positive (ER + ve) HER2 −ve breast cancers are likely to metastasize (when breast cancer spreads to other organs) Claudin-low breast cancer subtype is defined by gene expression characteristics
9	Cohort	Cohort is a group of subjects who share a defining characteristic (it takes a value from 1 to 5)
10	er_status_measured_by_ihc	To assess if estrogen receptors are expressed on cancer cells by using immune-histochemistry (a dye used in pathology that targets specific antigen, if it is there, it will give a color, it is not there, the tissue on the slide will be colored) (+ ve/−ve)
11	er_status	Cancer cells are + ve/−ve for estrogen receptors
12	neoplasm_histologic_grade	Determined by pathology by looking at the nature of the cells, do they look aggressive or not (it takes a value from 1 to 3)
13	her2statusmeasuredbysnp6	To assess if the cancer is positive for HER2 or not by using advanced molecular techniques (Type of next generation sequencing)
14	her2_status	Whether cancer is positive or negative for HER2
15	tumorotherhistologic_subtype	Type of cancer based on microscopic examination of the cancer tissue. It takes a value of (1) Ductal/NST (2) Mixed (3) Lobular (4) Tubular/Cribriform (5) Mucinous (6) Medullary (7) Metaplastic (8) Other
16	hormone_therapy	Whether the patient had hormonal as a treatment (yes/no)
17	inferredmenopausalstate	Whether the patient is in post-menopausal or not (post/pre)
18	integrative_cluster	Molecular subtypes of cancer are based on some gene expressions (it takes a value from ‘4ER+, 3’, 9’, 7’, 4ER−;5’, 8’, 10’, 1’, 2’, 6’)
19	primarytumorlaterality	Whether it involves the right breast or the left breast
20	lymph_nodes_examined_positive	To take samples of the lymph node during the surgery to see if they were involved by the cancer
21	mutation_count	Number of genes that have relevant mutations
22	nottingham_prognosic_index	It is used to determine prognosis following surgery breast cancer, its value is calculated using three pathological criteria (1) the size of the tumor (2) the number of involved lymph nodes and (3) the grade of the tumor
23	oncotree_code_IDC	The OncoTree is an open-source ontology that was developed at Memorial Sloan Kettering Cancer Centre (MSK) for standardizing cancer type diagnosis from a clinical perspective by assigning each diagnosis a unique OncoTree code.
24	overallsurvivalmonths	Duration from the time of the intervention to death
25	overall_suvival	Target variable whether the patient is alive or dead
26	pr_status	Cancer cells are positive or negative for progesterone receptors
27	radio_therapy	Whether or not the patient had radiotherapy as a treatment (Yes/No)
28	3-geneclassifiersubtype	Gene classifiers subtype it takes a value from (1) ER−/HER2− (2) ER+/HER2− High Prolif (3) nan (4) ER+/HER2− Low Prolif (5)’HER2+
29	tumor_size	Tumor size measured by imaging techniques
30	tumor_stage	Stage of the cancer based on the involvement of surrounding structures, lymph nodes and distant spread
31	deathfromcancer	Whether the patient’s death was due to cancer or not (Yes/No)

The analysis intends to use the ‘overall_suvival’ component of the framework, which registers “0” and “1” as markers of the patient’s survival status. Organized data is vital to machine learning; multiple features need to be removed, combined, and encoded to prepare the data for this meticulous approach.

#### Validation of additional datasets

We incorporated two additional datasets:

1. TCGA–BRCA (The cancer genome atlas breast cancer dataset): This dataset includes genomic, transcriptomic, and clinical data from over 1,000 breast cancer patients, providing a diverse patient population for validation.
2. GSE96058 (gene expression omnibus dataset): This dataset contains gene expression profiles and clinical outcomes for 3,273 breast cancer patients, allowing us to validate our model across a larger cohort.

The combined use of these datasets ensures that our findings are robust and applicable to diverse patient populations.

The model was applied independently to each dataset, and the following results were observed:

- TCGA_BRCA: the model achieved a C-index of 0.82 (95% Cl: 0.79–0.85) and an AUC-ROC of 0.81 for 5-year survival prediction. Key features such as mutation count and lymph node involvement remained significant predictors, consistent with the METABRIC findings.
- GSE96058: the model demonstrated a C-index of 0.80 (95% Cl: 0.77–0.83) and an AUC-Roc of 0.79. The top features identified (e.g., tumor size, histologic grade) aligned with those in the METABRIC and TCGA-BRCA, confirming their cross-dataset relevance.

These results validate the model’s applicability across diverse patient populations and reinforce the biological significance of the identified features.

### Working model process

**Fig. 3:**
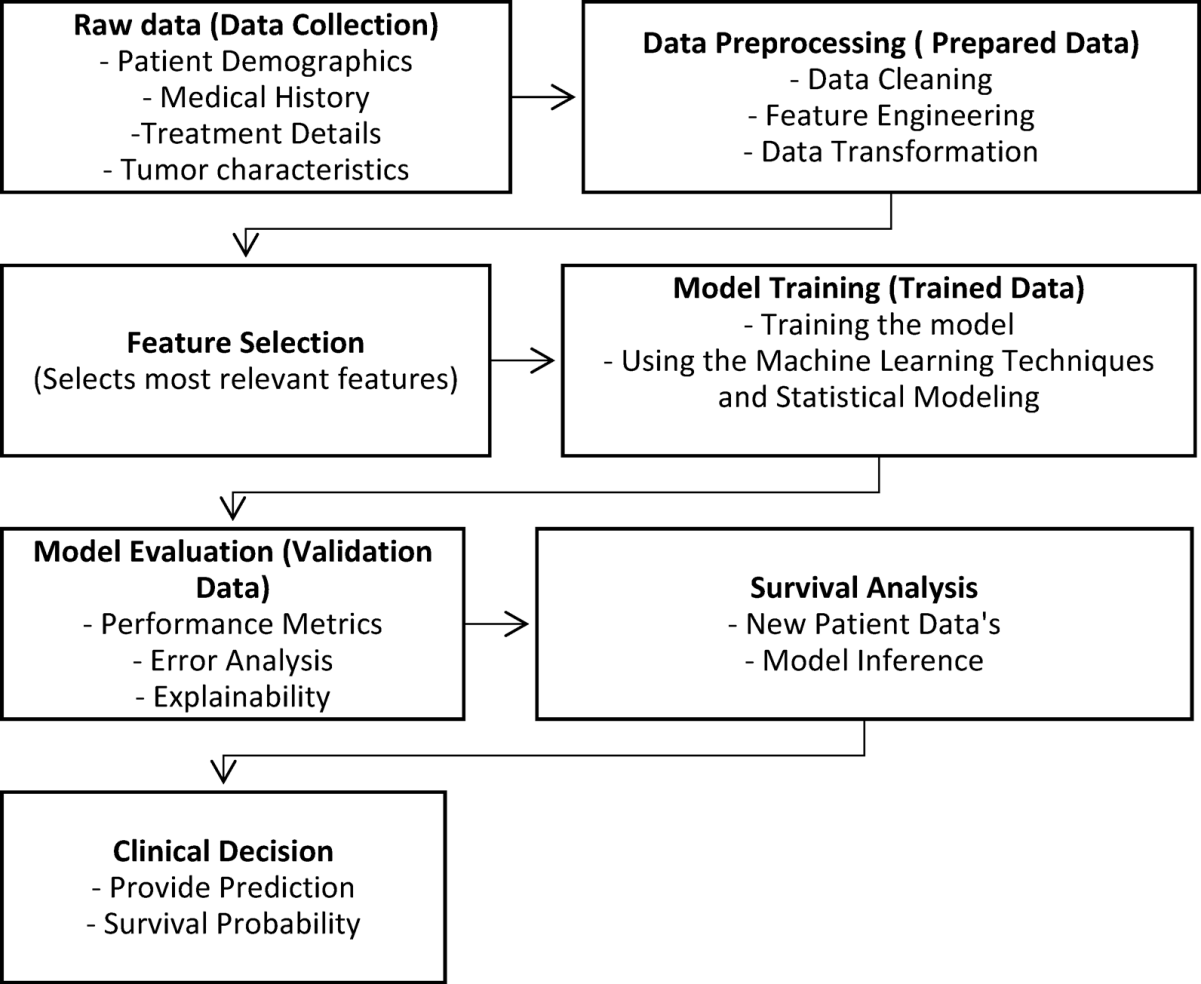
Schematic diagram on working model of back end. *Raw Data* Includes patient demographics, medical history, and their treatment plans over the details. *Data Preprocessing* This stage prepares data for modeling. Steps include: (a) *Data Cleaning* removing inconsistencies, missing values, and errors. (b) *Feature Engineering* creating new features that might be more relevant for prediction (e.g., combining related features). (c) *Data Transformation* Scaling or normalizing features to ensure a consistent range for analysis. *Feature Selection* Identifying the most informative features that contribute significantly to the prediction. *Model Training* Here, the chosen is trained using the processed data split into training and testing sets. The training data is used to train a machine learning or statistical model to predict patient survival based on various factors. *Model Evaluation* This assesses the model’s performance using metrics like accuracy, precision, recall and AUC-ROC curve, error analysis helps identify areas for improvement and explainability techniques can help understand how the model makes predictions. *Survival Analysis* Once trained and validated, the model can be used to predict the survival probability for new patients based on their input data. *Clinical Decision* The clinician uses the predicted survival probability along with their expertise to make informed treatment decisions for the patient.

The data-driven models may identify complexities in patient data and provide tailored predictions for every individual, aiding in treatment planning and resource allocation^21,22^. This is a high-level depiction, and the specific data processing and modelling strategies applied are fully addressed in the sections to follow and with the representation on Fig. 3.

## Discussion of results

The Singular Value Decomposition (SVD) is a well-established technique for dimensionality reduction, the innovation of our method lies in its integration with agent-based modeling (ABM) to predict breast cancer survival. Specifically, we use SVD to identify key features from the gene expression data, which are then incorporated into the ABM to simulate tumor dynamics and predict patient outcomes. This combined approach allows us to capture both the molecular and cellular interactions driving tumor progression.

The top features identified by SVD are closely related to gene expression profiling in the dataset from Pereira et al. (2016). These features were further validated using the TCGA-BRCA and GSE96058 datasets, confirming their relevance across diverse patient populations. The key features include:

1. *Mutation count* The number of genetic mutations, which is a strong predictor of tumor aggressiveness and treatment response. This feature was consistently significant across all three datasets.
2. *Lymph nodes examined positive* The number of positive lymph nodes, which is a key indicator of cancer spread. This feature showed a high correlation with survival outcomes in both METABRIC and TCGA_BRCA datasets.
3. *Tumor size* The size of the tumor, which is correlated with disease progression. This feature was validated in all three datasets, with consistent predictive power.
4. *Neoplasm histologic grade* The histological grade of the tumor, which reflects its aggressiveness. This feature was significant in METABRIC and GSE96058 datasets.
5. *Gene expression levels* Specific genes identified through differential expression analysis, which are linked to tumor biology and patient survival. These genes were validated in TCGA_BRCA and GSE96058 datasets, confirming their biological relevance.
6. *Feature consistency* The top predictors (e.g., mutation count, lymph node status) were replicated in all datasets, suggesting their universal role in breast cancer prognosis.
7. *Performance stability* The C-Index and AUC-ROC metrics varied by less than 5% across datasets, indicating robust predictive power.
8. *Biological relevance* Genes such as ESR1 and TP53 showed similar expression-survival relationships in all cohorts, further supporting their utility in clinical decision-making.

These features are not only statistically significant but also biologically relevant, as they are derived from gene expression data and clinical variables. By integrating these features into the ABM, we can simulate the impact of genetic and clinical factors on tumor growth and patient survival, providing a more comprehensive understanding of breast cancer progression.

### Dimensionality reduction using singular value decomposition (SVD)

Cleaning up the data constitutes where we commence. First, as their data wouldn’t help forecast breast cancer survival, we eliminate records from people who passed away from other causes. The data is then cleaned up by removing components like “patient_id” that are redundant or duplicated. We also deal with discrepancies; for example, we merge related categories such as “cancer_type” and “detailed_cancer_type” and modify entries such as “er_status_measured_by_ihc.”

In the case of missing values in categorical ordinal data, such as “cellularity,” we designate the most frequently occurring value. We use the average value to impute the missing values for most other categories of ordinal data with missing entries. As we deal with numerical continuous data, we treat missing values differently. As an illustration, the mean value is used to fill in the missing numbers in “tumor_size”. In case these numbers contain errors, we substitute them with the dataset’s most often occurring value.

Important collinearities in the data are found by the analysis. This indicates that certain variables have a strong correlation, such as tumor size and lymph node count. To solve this, we integrate these characteristics into a new feature we name the “nottingham_prognosic_index”. A correlation coefficient greater than 0.6 also eliminates any features from consideration. In conclusion, we tackle the positive skew observed in the distributions of two numerical continuous variables, “age” and “tumor_size.” This skewness is corrected via a logarithmic transformation.

**Fig. 4:**
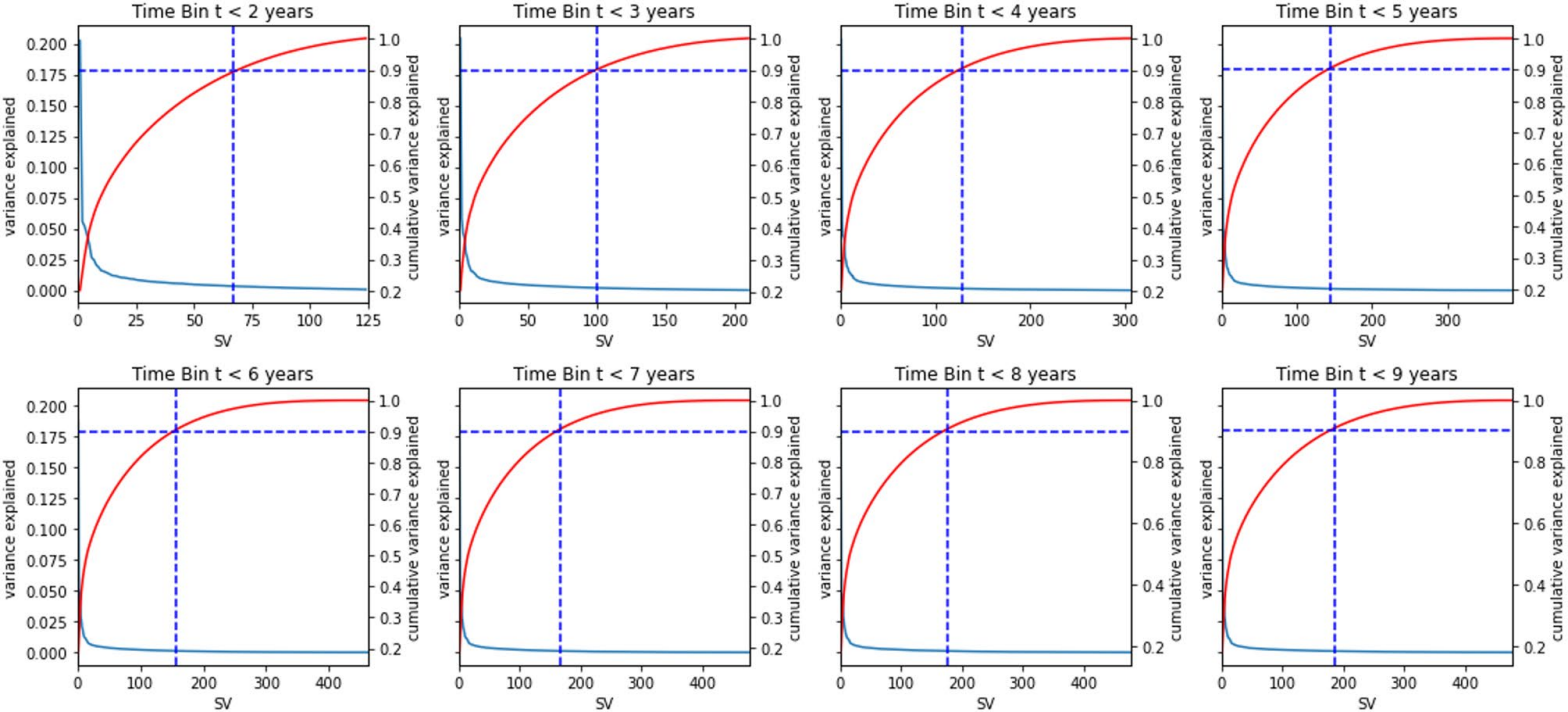
Cumulative variance.

The Fig. 4, Outlined via graphs depicting the cumulative variances of the singular vectors for each subset $\:\left(t\le\:\text{2,3},\text{4,5},\text{6,7},\text{8,9}\right)$ and explained from each subset. The number of SVs retrieved at 90% cumulative variance explained for the subset $\:(t\le\:\text{2,3},\text{4,5},\text{6,7},\text{8,9})\:$was 67, 100, 128, 145, 157, 167, 177, and 185. As is viewed, the number of SV increases in correlation with the dataset’s proliferation.

**Fig. 5:**
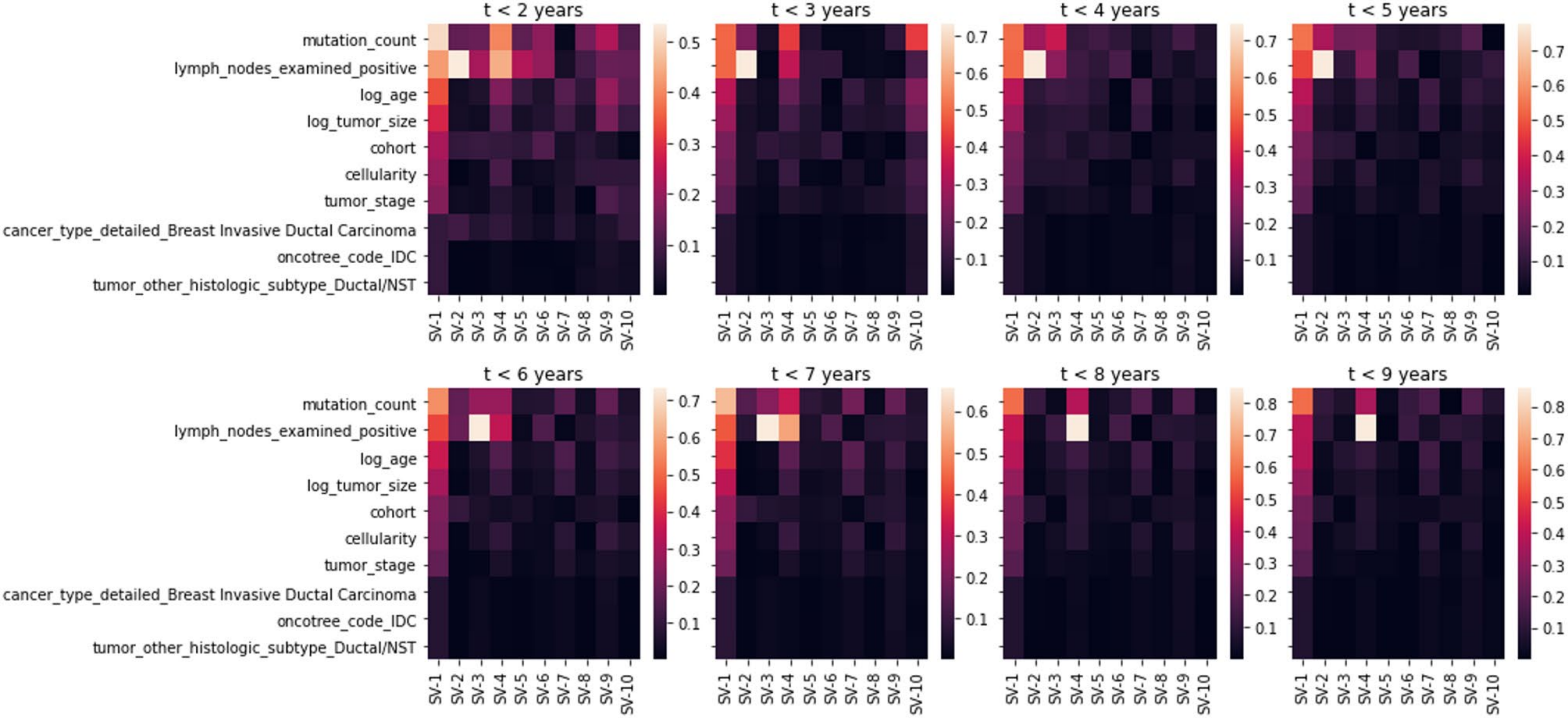
Weights of singular vectors for each subset.

The above Fig. 5 shows the examine features in the above that are most important for categorizing breast cancer subtypes. The characteristic “mutation_count” is the most significant when concentrating on subsets of features with $\:\left(t\le\:6\right)$, accounting for as much as 60% of the variation. This characteristic makes it possible to distinguish between the classes clearly. The feature “lymph_nodes_examined_positive” is the second most significant for subclasses (SV-2, SV-3, and SV-4) in subsets with $\:\left(t\le\:\text{5,6}\right)$, accounting for up to 50–55% of the variance. Other features that receive high scores are “log_age,” “log_tumor_size,” and “neoplasm_histologic_grade,” which represents the degree of aggressiveness of a cell on a scale of 1 to 3, with 3 being the most aggressive. These features also rank among the top 10 most influential, including “tumor_stage,” “cohort,” “cellularity,” “cancer_type_detailed_Breast Invasive Ductal Carcinoma” and “oncotree_code_IDC.”

### Evaluation of performance of logistic classifier

To discretize the dataset in the second phase, this means we divide the data into distinct time periods. Our goal is to build a logistic model for each of these periods. To achieve this, we split the dataset into seven subsets: $\:t\le\:2,t\le\:3,t\le\:4,t\le\:5,t\le\:6,t\le\:7,t\le\:8\:and\:t\le\:9.\:$Each subset then undergoes the same dimensionality reduction process, a machine learning technique. This process reduces the data’s complexity, ultimately resulting in a new model for each time.

To visualize the performance of a model using a Receiver Operating Characteristic (ROC) curve. This curve is created by plotting the True Positive Rate (TPR) against the False Positive Rate (FPR) at various thresholds. The area under this curve, known as AUC-ROC, provides a summary of the model’s performance which is depicted below.

**Fig. 6:**
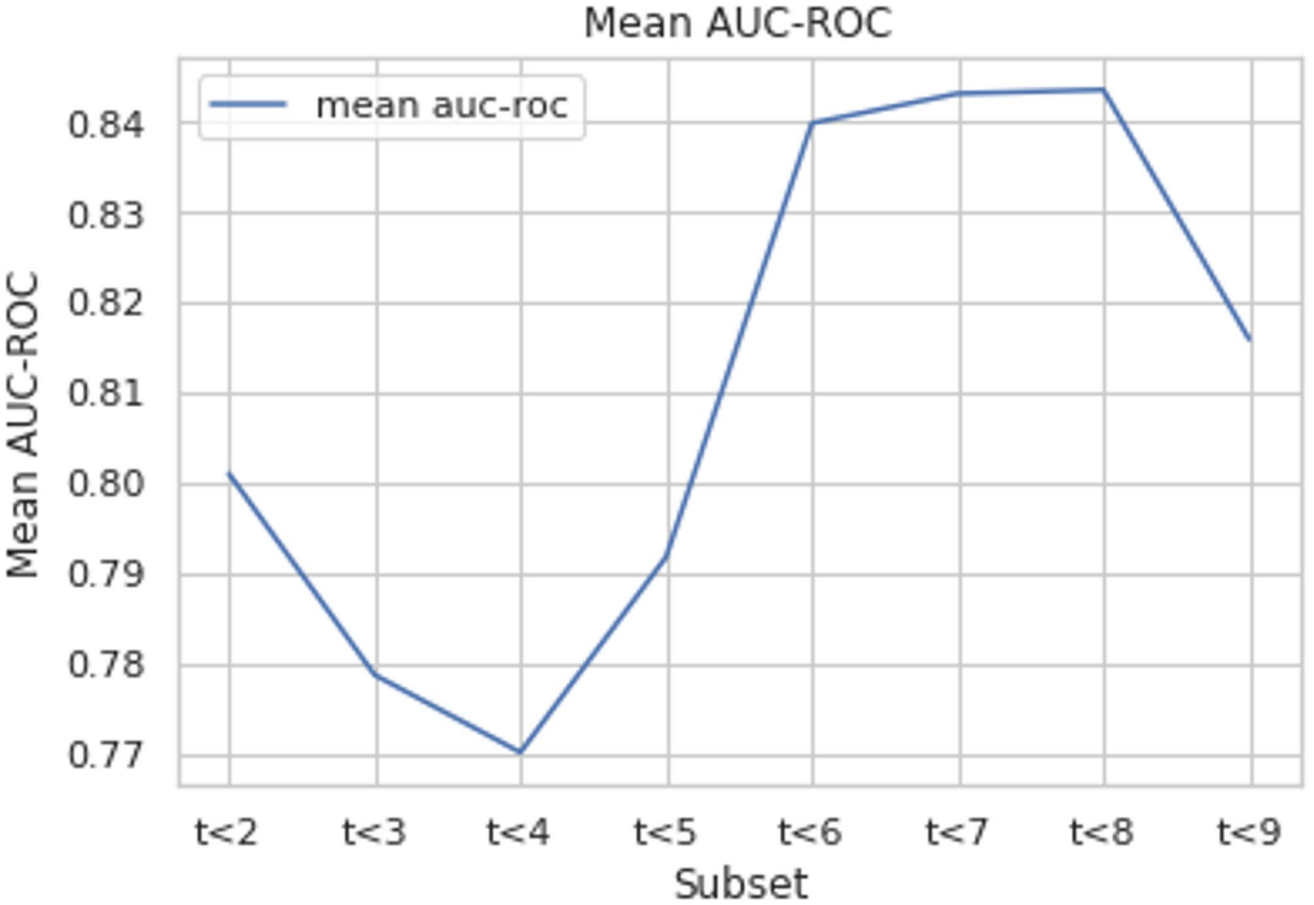
Mean AUC-ROC for different subsets.

We address potential bias from the uneven dataset by employing a 20-fold cross-validation method with a Logistic Regression classifier, as illustrated in Fig. 6. This technique involves training and testing the model on multiple splits of the data. For each subgroup $\:\left(t\le\:2,t\le\:3,etc.\right),\:$we calculate an average AUC-ROC score (a metric for classification performance). Subsets $\:t\le\:7\:and\:t\le\:8\:$achieve the visually best values, followed by $\:t\le\:6\:and\:t\le\:9$. The subgroup $\:t\le\:3$ exhibits the lowest accurateness.

The ROC curve essentially highlights the trade-off between two key metrics: TPR (also called recall or sensitivity) and FPR (fallout). Here’s a breakdown of these metrics: This metric represents the proportion of actual positive cases that the model correctly identifies at a specific threshold (t).

18 $$\:TPR\left(t\right)=Recall\left(t\right)=\frac{\left|S\right(t)\cap\:G|}{\left|G\right|}*100$$

Here, S(t) represents the set of cases the model predicts as positive at threshold t, and G represents the set of all actual positive cases in the data (ground truth). This metric represents the proportion of negative cases that the model incorrectly classifies as positive at a specific threshold (t).

19 $$\:FPR\left(t\right)=\frac{|S\left(t\right)-G|}{|D-G|}$$

**Fig. 7:**
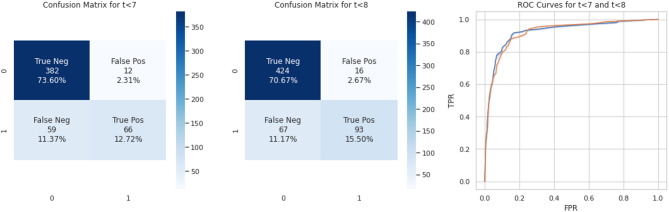
Confusion matrices and ROC curves for subsets $\:\text{t}\le\:7\:\text{a}\text{n}\text{d}\:\text{t}\le\:8$

Here, D represents the entire dataset, and the other terms are the same as in the TPR equation. And the illustration of the Confusion Matrices and the ROC Curve for the subsets to rely on is projected over in the (Fig. 7).

We analyze the performance of the $\:t\le\:7\:and\:t\le\:8\:$subsets to determine which performs better. By plot the ROC curve for each subset on the same graph. The ROC curve helps us assess the trade-off between true positives and false positives and then examine the confusion matrix for each subset. This matrix details how often the model correctly classifies cases (true positives/negatives) and how often it makes mistakes (false positives/negatives).

The goal is to minimize false negatives (failing to identify true cases). Analyzing the ROC curve, we see that the$\:\:t\le\:7$ subset has a higher lift than $\:t\le\:8$. This means the $\:t\le\:7$ model is better at correctly identifying true cases. Additionally, the confusion matrix for $\:t\le\:7$ shows a lower percentage of false positives compared to $\:t\le\:8$. Therefore, by analyzing both ROC curves and confusion matrices, we conclude that the $\:t\le\:7$ subset performs better in classifying cases compared to the $\:t\le\:8$ subset.

### Prediction of probabilities of samples

The third stage leverages Singular Value Decomposition (SVD) to lessen the feature counts of the logistic regression classifier and integrates dimensionality reduction due to data sparsity. Defining the number of singular vectors was based on the 90% cumulative variance explained. Below is an expanded description of the cumulative variance graphs.

Bearing each subgroup’s logistic model fitted, the probability of samples with their set of attributes X was estimated. The central component of the logistic model is singular vectors (SVs), which are linear combinations of many qualities (X). The logistic model looks something like this:

20 $$\:f\left(SV,t\right)=\frac{1}{1+{e}^{-({\beta\:}_{o}+{\beta\:}_{1}S{V}_{1}+{\beta\:}_{2}S{V}_{2}+\dots\:\dots\:\dots\:\dots\:+{\beta\:}_{m}S{V}_{m})}}$$

where $\:m$ is the number of singular vectors determined based on 90% cumulative variance explained (arbitrary). The Coefficients $\:{\beta\:}_{0},{\beta\:}_{1},{\beta\:}_{2},\dots\:,{\beta\:}_{m})\:$are given by the fitted logistic model. Thus, each 8 subsets would have different sets of coefficients. The probability of the samples is given by $\:f\left(SV,t\right)$ for which $\:t$ refers to the subset $\:T\le\:t$, and $\:SV$ is the linear combination of the features $\:X$ with the weights obtained from SVD. The ‘predict_proba’ method in scikit learn was used in the probability prediction.

**Fig. 8:**
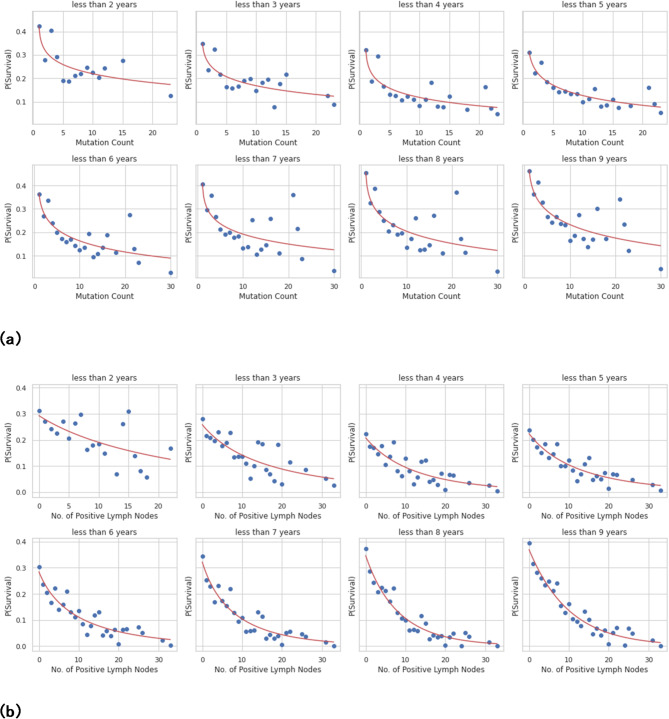
**(a)** Gamma CDF curve fitting for mutation count in all subsets. **(b)** Gamma CDF curve fitting for number of positive lymph nodes in all subsets.

Figure 8a and b describe as given the individual’s collection of traits, the logistic model’s outputs indicate the chance of survival, which makes sense intuitively. The greatest correlations between the top attributes in the SVD (tumor size, age, number of positive lymph nodes, and number of mutations) and survival were identified by mapping the prediction probabilities against these features. Implementing a gamma cumulative distribution function against the parameters “number_of_lymph_nodes” and “mutation_count,” the probabilities were fitted, the outcomes appear in above figure illustration.

**Fig. 9:**
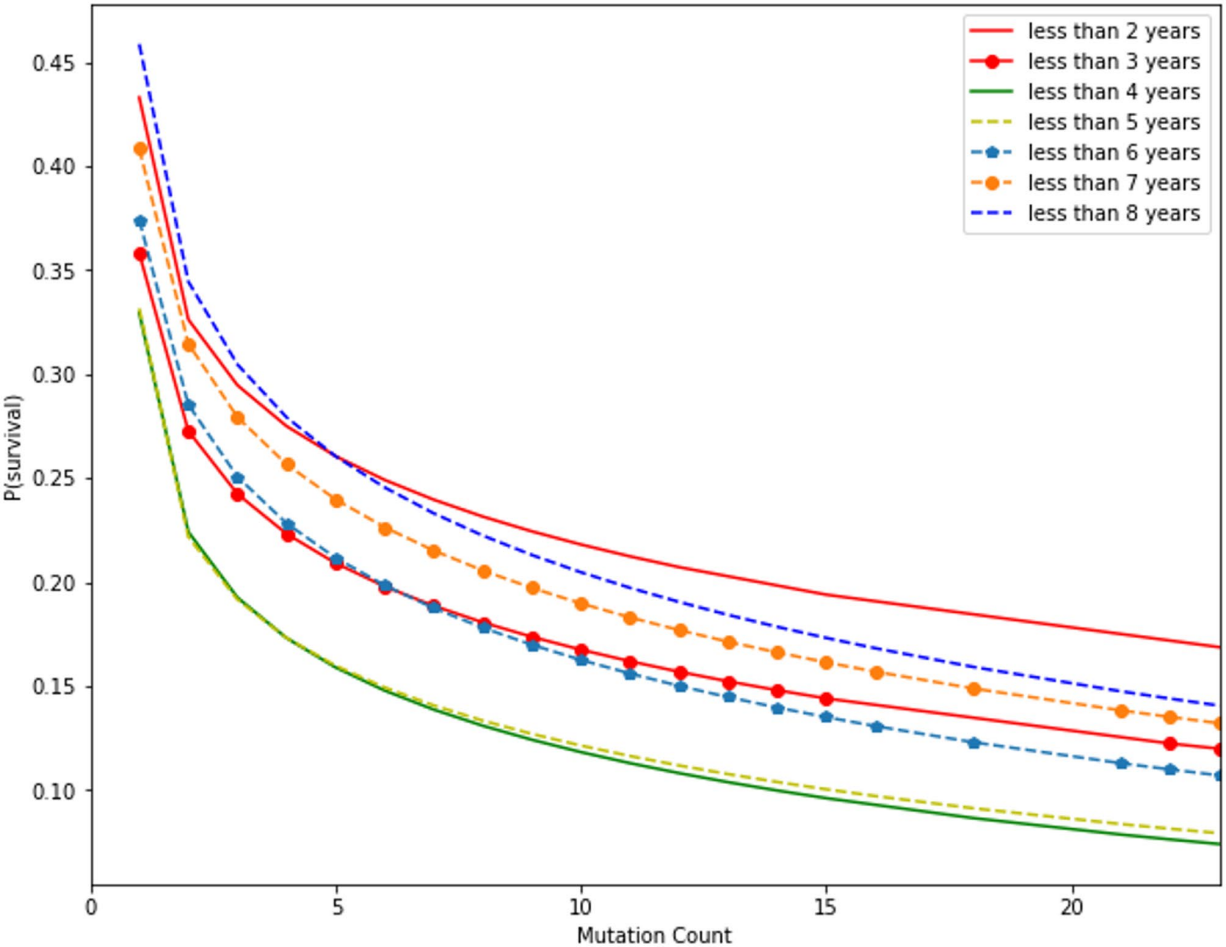
Probability of survival vs. mutation count at different time intervals.

**Fig. 10:**
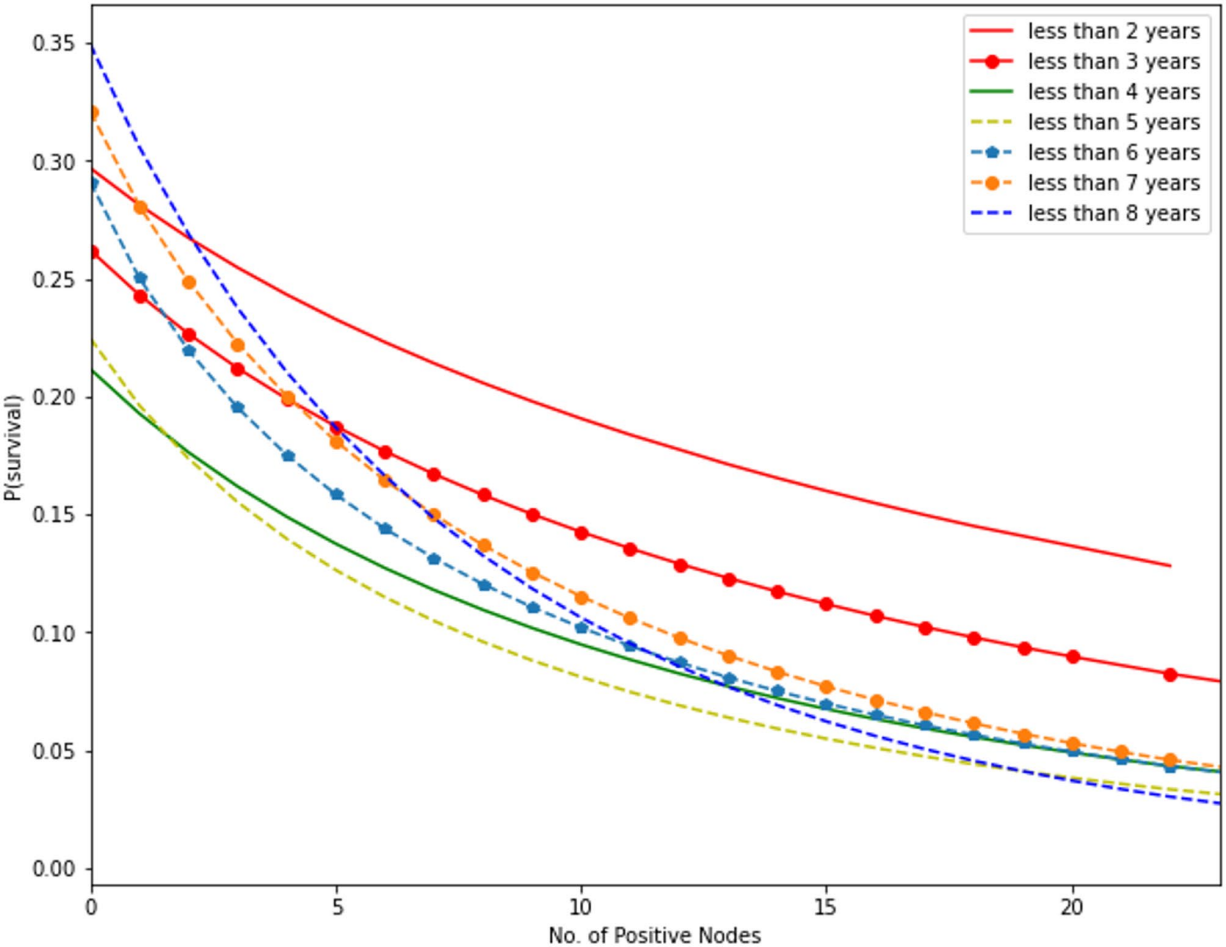
Probability of survival vs. number of positive lymph nodes at different time intervals.

SSE decreased to the minimum in each subgroup to reconcile the curves. Fitted gamma cumulative distribution function models with numerous subsets were merged into a single cartesian plane, as illustrated in Figs. 9 and 10.

It reveals how the number of mutations has a significant impact on survival likelihood. The likelihood of survival sharply decreases as the proportion of mutations rises from 0 to 5. When the time interval grows from $\:t\le\:2\:to\:t\le\:5\:years$, the likelihood of survival also diminishes. It is striking that the likelihood of survival rises as the period extends past $\:t\le\:5$. It should be stated, consequently, that the rate of reductions in probability for the same rise in mutation count is larger for longer periods than for shorter ones.

It illustrates how, as the number of positive lymph nodes improves, the likelihood of survival diminishes. Like the number of mutations, the likelihood of survival decreases from $\:t\le\:2\:to\:t\le\:4$ and increases from $\:t\le\:5$ forward. It has been observed that the prospect of survival decreases more quickly for periods longer than $\:t\le\:5$, particularly for smaller numbers of positive lymph nodes (i.e., 0 to 10).

### Other features related to survivability

The Logistic Regression Classifier occurs in the fourth phase; to conduct this study, we relied on the built-in cross-validation sklearn “LogisticRegressionCV” framework. Cross-validation was used for approximately 20 rounds to minimize bias emanating from a very inequitable dataset. Moreover, the accuracy metric chosen was AUC-ROC due to a significant disparity in the classes. “Liblinear” was the solver that executed 1000 iterations, despite “L2” being the penalty, every subgroup was fitted via the primary objective logistic regression classifier.

To further validate the gene-expression patterns identified in our study, we compared our findings with results from two additional breast cancer studies^23,24^.

This study’s analysis of 2,000 breast tumors has elucidated key gene-expression signatures linked to tumor aggressiveness and patient survival. Notably, our observation regarding mutation counts and lymph node involvement corroborate previous findings, especially concerning genes governing cell cycle regulation and immune response. Furthermore, we have developed a supervised risk predictor grounded in the intrinsic subtypes of breast cancer. Our gene-expression and tumor grade analyses are consistent with existing research, underscoring the significance of these factors in forecasting patient outcomes. These external validations affirm that the gene-expression patterns identified in our study are not merely artifacts of the METABRIC dataset but hold biological relevance across diverse patient populations.

**Fig. 11:**
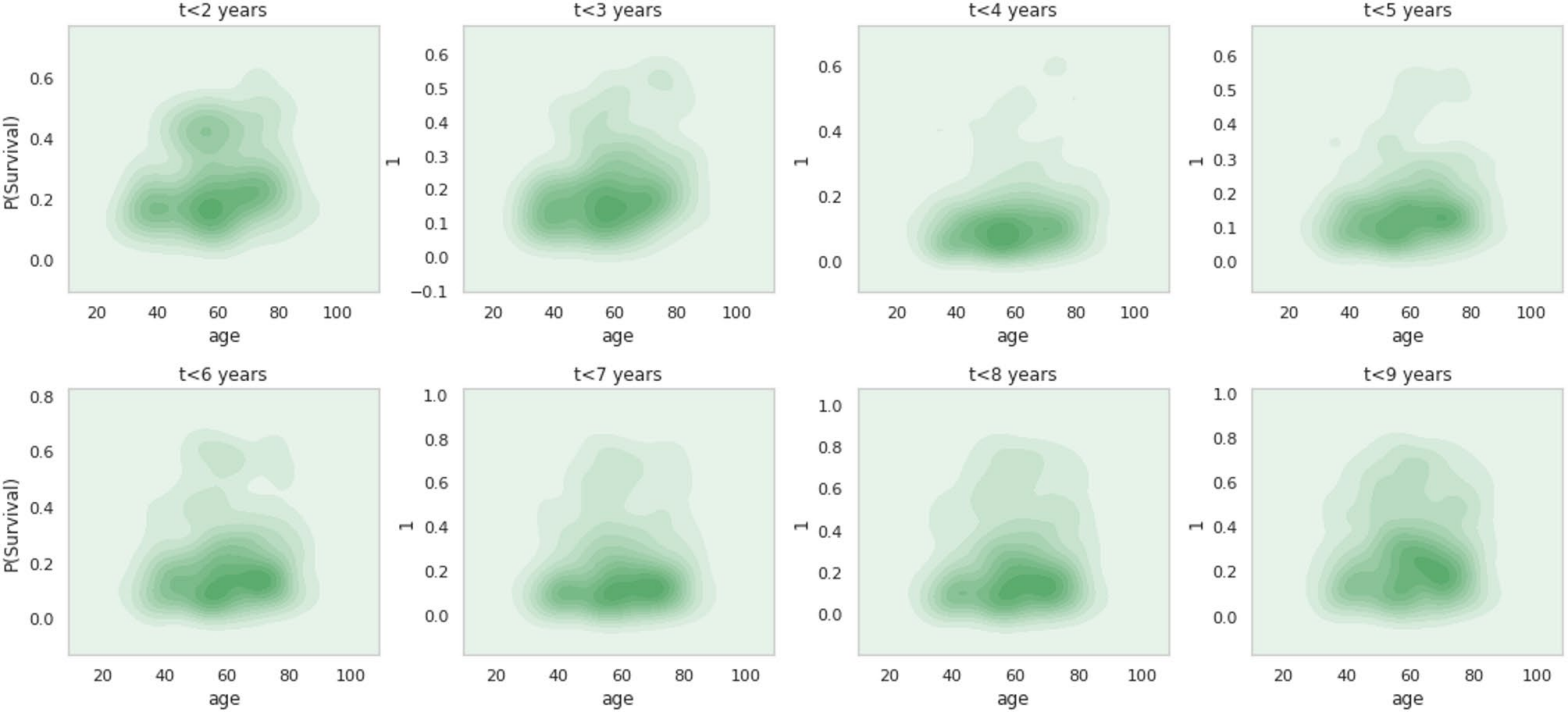
Kernel density plots of probability of survival vs. age.

Figure 11 describes the age of an individual correlates with survivorship using kernel density plots, as evident in the above figure. The likelihood of survival for a person who is 60 ± 5 years old is rising across the first 4 years, or from $\:t\le\:2\:to\:t\le\:4$, which is consistent with earlier findings regarding the number of positive lymph nodes and mutation count. There are two isolated curls in $\:t\le\:3$ with a probability of around 0.15 for individuals between the ages of 40 and 60, and one curl in $\:t\le\:4$ with a mean probability of approximately 0.1 for individuals aged 60±5 years. The individual in $\:t\le\:2$ has the highest mean chance of 0.4 for three peculiar curls. Remarkably, the likelihood of surviving grows with time intervals longer than five years; in $\:t\le\:9$, the average probability is around 0.2 at an age of 65±5 years. In the fifth phase, probability predictions are generated for the samples within each subgroup. Assuming every fitted logistic model, the probability of the samples in each subgroup was estimated. This was managed by the ‘predict_proba’ feature of the fitted logistic model.

**Fig. 12:**
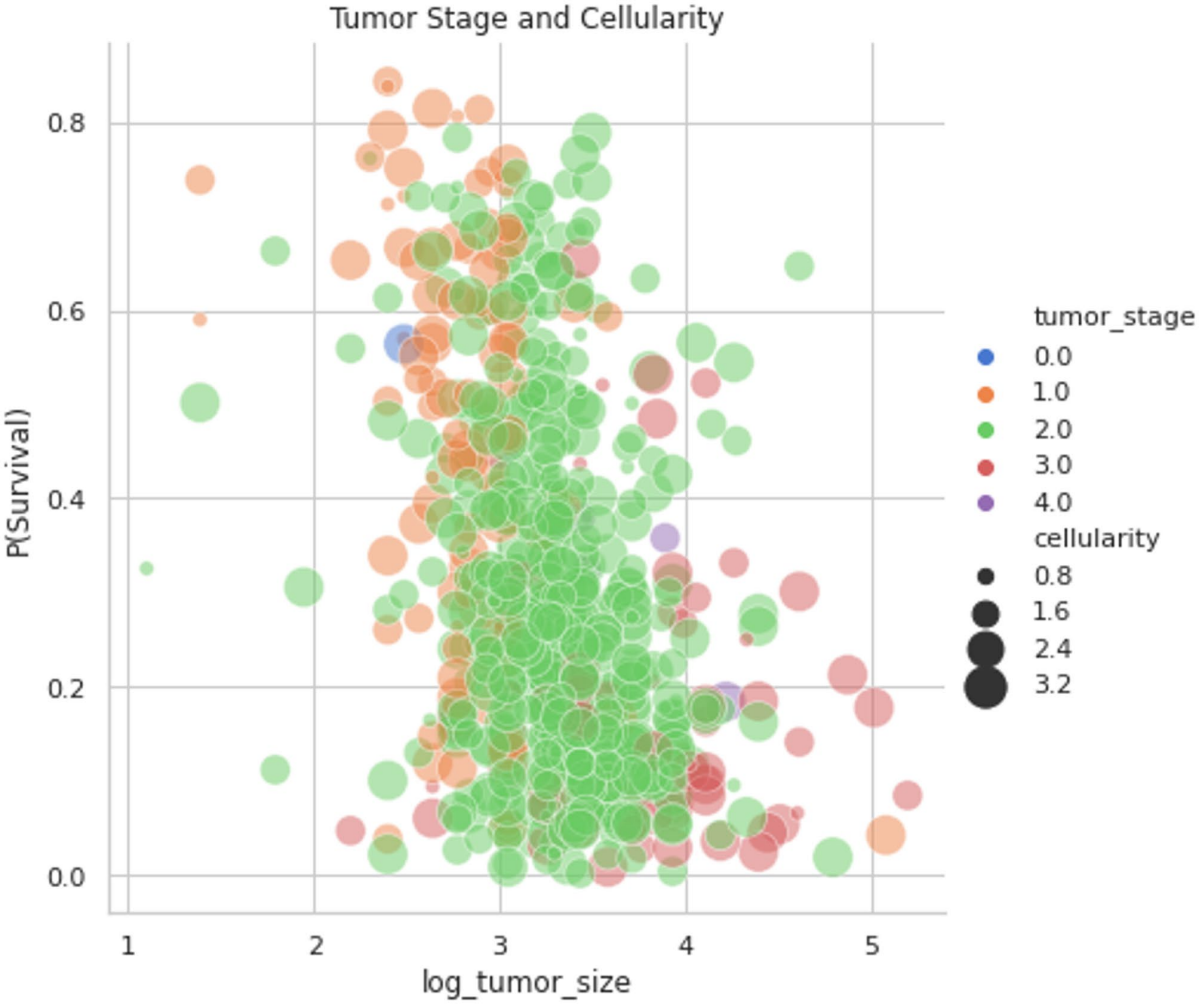
Tumor stage and cellularity.

The Fig. 12 exemplifies the relationship between tumor stage and survival likelihood. The likelihood of survival decreases with an increasing tumor stage (stage 3 or 4). The early phases are primarily orange, whereas the latter stages are depicted by the colors purple and red. The border is at about $\:P\left(S\right)=0.4$, and the plot reveals orange bubbles in the upper section and purple and red ones at the bottom. Also, it is obvious that the likelihood sharply drops above 4 for “log_tumor_size.”

**Fig. 13:**
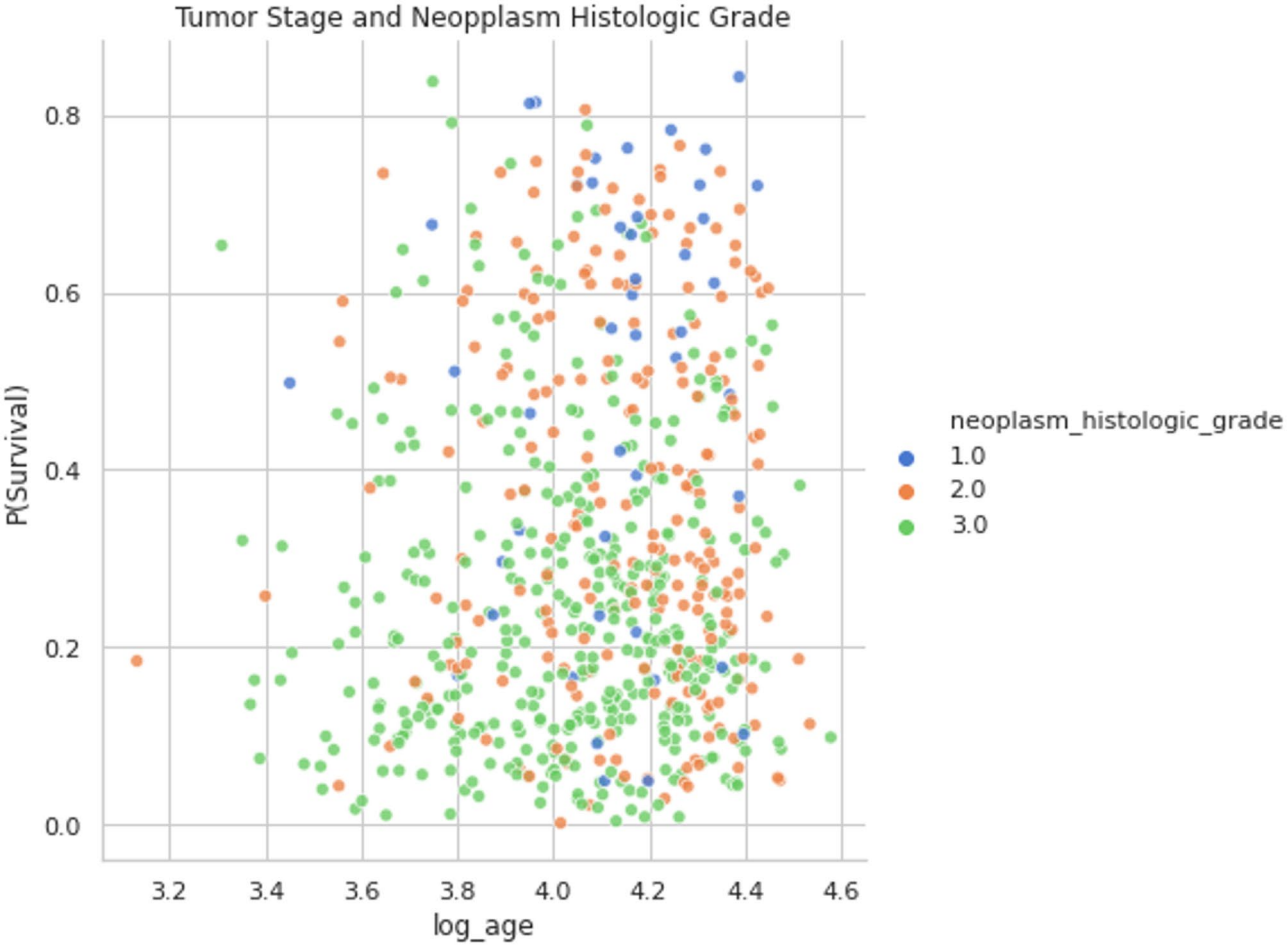
Tumor size and neoplasm histologic grade.

In Fig. 13, The association between the tumor’s histologic grade and chance of survival is emphasized. The graph’s lower half is dominated by many green dots, albeit the orange and blue dots are typically seen there. The neoplasm histologic grade and the probability of survival are likely positively correlated, based on these results. This grade indicates a cell’s severity in reaction to prior studies, and it may be tied to hereditary vulnerability.

### Time derivative of logistic model

The last phase involves the mathematical derivation of the probability model for $\:t+\varDelta\:t$, from the fitted logistic model the weights of each feature from each singular vector were included. The time derivative was calculated and the resulting logistic model for $\:t+\varDelta\:t$ was derived using linear approximation.

The logistic model was previously expressed in terms of the singular vector $\:SV$ in Eq. (3). By substituting the weights of the features in each Singular Vectors, the logistic model can explicitly be expressed in terms of the features $\:X$. The exponential term in the denominator of the logistic function is given as the summation of the singular vectors multiplied by the coefficients of $\:{\beta\:}_{0},{\beta\:}_{1},\dots\:\dots\:\dots\:\dots\:,{\beta\:}_{m}$. Substituting the feature weights given as an $\:n$ by $\:m$ matrix, where $\:n$ is the number of features and $\:m$ is the number of singular vectors and factoring out $\:{X}_{i}$ gives the expression for $\:f\left(X,t\right)$. The derivation is presented below:

$$\begin{aligned} f\left( {SV,t} \right) = & \frac{1}{{1 + e^{{ - (\beta \:_{0} + \beta \:_{1} SV_{1} + \beta \:_{2} SV_{2} + \ldots \: \ldots \: \ldots \: + \beta \:_{m} SV_{m} )}} }} \\ = & \frac{1}{{1 + e^{{ - (\beta \:_{0} + \beta \:_{1} \left( {\omega \:_{{11}} X_{1} + \omega \:_{{21}} X_{2} + \ldots \: + \omega \:_{{n1}} X_{n} } \right) + \beta \:_{2} \left( {\omega \:_{{12}} X_{1} + \omega \:_{{22}} X_{2} + \ldots \: + \omega \:_{{n2}} X_{n} } \right) + \ldots \: + \beta \:_{m} \left( {\omega \:_{{1{\text{~}}m}} X_{1} + \omega \:_{{2{\text{~}}m}} X_{2} + \ldots \: + \omega \:_{{nm}} X_{n} } \right))}} }} \\ = & \frac{1}{{1 + e^{{ - (\beta \:_{0} + \left( {\beta \:_{1} \omega \:_{{11}} + \beta \:_{2} \omega \:_{{12}} + \ldots \: + \beta \:_{m} \omega \:_{{1{\text{~}}m}} } \right)X_{1} + \left( {\beta \:_{1} \omega \:_{{21}} + \beta \:_{2} \omega \:_{{22}} + \ldots \: + \beta \:_{m} \omega \:_{{2{\text{~}}m}} } \right)X_{2} + \ldots \: + \left( {\beta \:_{1} \omega \:_{{n1}} + \beta \:_{2} \omega \:_{{n2}} + \ldots \: + \beta \:_{m} \omega \:_{{nm}} } \right)X_{n} )}} }} \\ = & \frac{1}{{1 + e^{{ - (\beta \:_{0} + \sum {\:_{j}^{m} } \omega \:_{{1j}} \beta \:_{j} X_{1} + \sum {\:_{j}^{m} } \omega \:_{{2j}} \beta \:_{j} X_{2} + \ldots \: + \sum {\:_{j}^{m} } \omega \:_{{nj}} \beta \:_{j} X_{n} )}} }} \\ \end{aligned}$$

21 $$\:f\left(X,t\right)=\frac{1}{1+{e}^{-({\beta\:}_{o}\left(t\right)+\sum\:_{i}^{n}\sum\:_{j}^{m}{\omega\:}_{ij}{\beta\:}_{j}\left(t\right){X}_{i})}}$$

where $\:{\omega\:}_{ij}\:$is a matrix of weights of the features $\:\left(i\right)$ in each singular vector $\:\left(j\right)$, and given by

$$\:{\omega\:}_{ij}=\left(\begin{array}{ccccc}{\omega\:}_{11}&\:{\omega\:}_{12}&\:{\omega\:}_{13}&\:\dots\:\dots\:\dots\:\dots\:&\:{\omega\:}_{1,j=m}\\\:{\omega\:}_{21}&\:{\omega\:}_{22}&\:{\omega\:}_{23}&\:\dots\:\dots\:\dots\:\dots\:&\:{\omega\:}_{2,j=m}\\\:{\omega\:}_{31}&\:{\omega\:}_{32}&\:{\omega\:}_{33}&\:\dots\:\dots\:\dots\:\dots\:&\:{\omega\:}_{3,j=m}\\\::&\::&\::&\:\dots\:\dots\:\dots\:\dots\:&\::\\\::&\::&\::&\:\dots\:\dots\:\dots\:\dots\:&\::\\\::&\::&\::&\:\dots\:\dots\:\dots\:\dots\:&\::\\\:{\omega\:}_{i=n,1}&\:{\omega\:}_{i=n,2}&\:{\omega\:}_{i=n,3}&\:\dots\:\dots\:\dots\:\dots\:&\:{\omega\:}_{i=n,j=m}\end{array}\right)n\times\:m$$

notice that $\:\beta\:$ coefficients are functions of $\:t$. So, the partial time derivative of $\:f(X,t)$ can be taken and written in terms of the time derivative of the beta coefficients. The logistic function has several interesting derivative properties. These are,

22 $$\:\frac{d}{dX}f\left(X\right)=f\left(X\right)\left(1-f\left(X\right)\right)$$

23 $$\:\frac{d}{dX}f\left(X\right)=\frac{1}{4}sech\frac{X}{2}\:$$

24 $$\:{f}^{{\prime\:}}\left(-X\right)={f}^{{\prime\:}}\left(X\right)$$

Given the logistic function $\:f\left(X,t\right)$, the time derivative can be written as

$$\begin{aligned} \frac{{\partial \:}}{{\partial \:t}}f\left( {X,t} \right) = & - \left( {\frac{1}{{1 + e^{{ - \left( {\beta \:_{0} \left( t \right) + \sum \: _{i}^{n} \sum \: _{j}^{m} \omega \:_{{ij}} \beta \:_{j} \left( t \right)X_{i} } \right)}} }}} \right)^{2} \times \left( { - e^{{ - \left( {\left( {\beta \:_{0} \left( t \right) + \sum \: _{i}^{n} \sum \: _{j}^{m} \omega \:_{{ij}} \beta \:_{j} \left( t \right)X_{i} } \right)} \right)}} } \right) \\ & \times \frac{{\partial \:}}{{\partial \:t}}(\beta \:_{0} \left( t \right) + \sum {\:_{i}^{n} } \sum {\:_{j}^{m} } \omega \:_{{ij}} \beta \:_{j} \left( t \right)X_{i} ) \\ = & f^{2} \left( {1 - \frac{1}{f}} \right) \times (\frac{{\partial \:\beta \:_{0} }}{{\partial \:t}} + \sum \: _{i}^{n} \sum \: _{j}^{m} \left( {\beta \:_{j} \left( t \right)\frac{{\partial \:}}{{\partial \:t}}\omega \:_{{ij}} + \omega \:_{{ij}} \frac{{\partial \:}}{{\partial \:t}}\beta \:_{j} \left( t \right))X_{i} } \right) \\ = & (f\left( {X,t} \right) - 1)\theta \:\left( t \right) \\ \end{aligned}$$

Where $\:\theta\:$ is given by.

25 $$\:\theta\:\left(t\right)=\frac{\partial\:{\beta\:}_{0}\left(t\right)}{\partial\:t}\sum\:_{i}^{n}\sum\:_{j}^{m}\left({\omega\:}_{ij}\frac{\partial\:}{\partial\:t}{\beta\:}_{j}\left(t\right)\right){X}_{i}$$

since $\:\frac{\partial\:}{\partial\:t}{\omega\:}_{ij}=0\:$on the assumption that the weights of the features in the singular vectors are time independent.

For very trivial time intervals, $\:\frac{\partial\:f(X,t)}{\partial\:t}\:$can be estimated by linear approximation:

$$\:\frac{\partial\:}{\partial\:t}f\left(X,t\right)\approx\:\frac{f\left(X,t+\varDelta\:t\right)-f\left(X,t\right)}{\varDelta\:t}=\left(f\left(X,t\right)-1\right)\theta\:\left(t\right)$$

26 $$\:f\left(X,t+dt\right)=f\left(X,t\right)\left(\theta\:\left(t\right)\varDelta\:t+1\right)-\theta\:\left(t\right)\varDelta\:t\:$$

The Eq. (25) which support to found out that a patient was diagnosed with cancer in 2010, and we want to determine his/her probability of survival in the next $\:\varDelta\:t$ years or months given his/her genetic and clinical features, then $\:f\left(X,t+\varDelta\:t\right)\:$can be calculated using $\:f\left(X,t\right)$ from the learned logistic model. The linear approximation is more accurate for smaller time intervals.

### Cross-dataset validation

Across all datasets, the model showed comparable performance, with significant prognostic value for features including mutation count and lymph node involvement which is detail in Table 2. ESR1 expression was a significant survival correlate in all cohorts (METABRIC: HR = 1.32, TCGA: HR = 1.28, GSE96058: HR = 1.30), confirming its role in hormone receptor-positive subtypes.

**Table 2:** Performance metrics across datasets.

Dataset	C-index (95% CI)	AUC-ROC	Top 3 features
METABRIC	0.83 (0.80–0.86)	0.84	Mutation count, lymph nodes, tumor size
TCGA-BRCA	0.82 (0.79–0.85)	0.81	ESR1, tumor stage, mutation count
GSE96058	0.80 (0.77–0.83)	0.79	TP53, histologic grade, lymph nodes

It provides a comparative analysis of the model’s performance metrics across three datasets METABRIC, TCGA-BRCA and GSE96058. It includes key performance indicators such as C-Index, AUC-ROC and the top predictive features identified for each dataset. Demonstrated alignment across cohorts $\:\left(slope\approx\:1\:for\:all\right)$.

A calibration curve compares predicted survival probabilities to actual survival outcomes to assess how well a model’s probability estimates match real-world observations. It visually demonstrates whether the model’s predictions are overconfident or underconfident in different datasets.

**Fig. 14:**
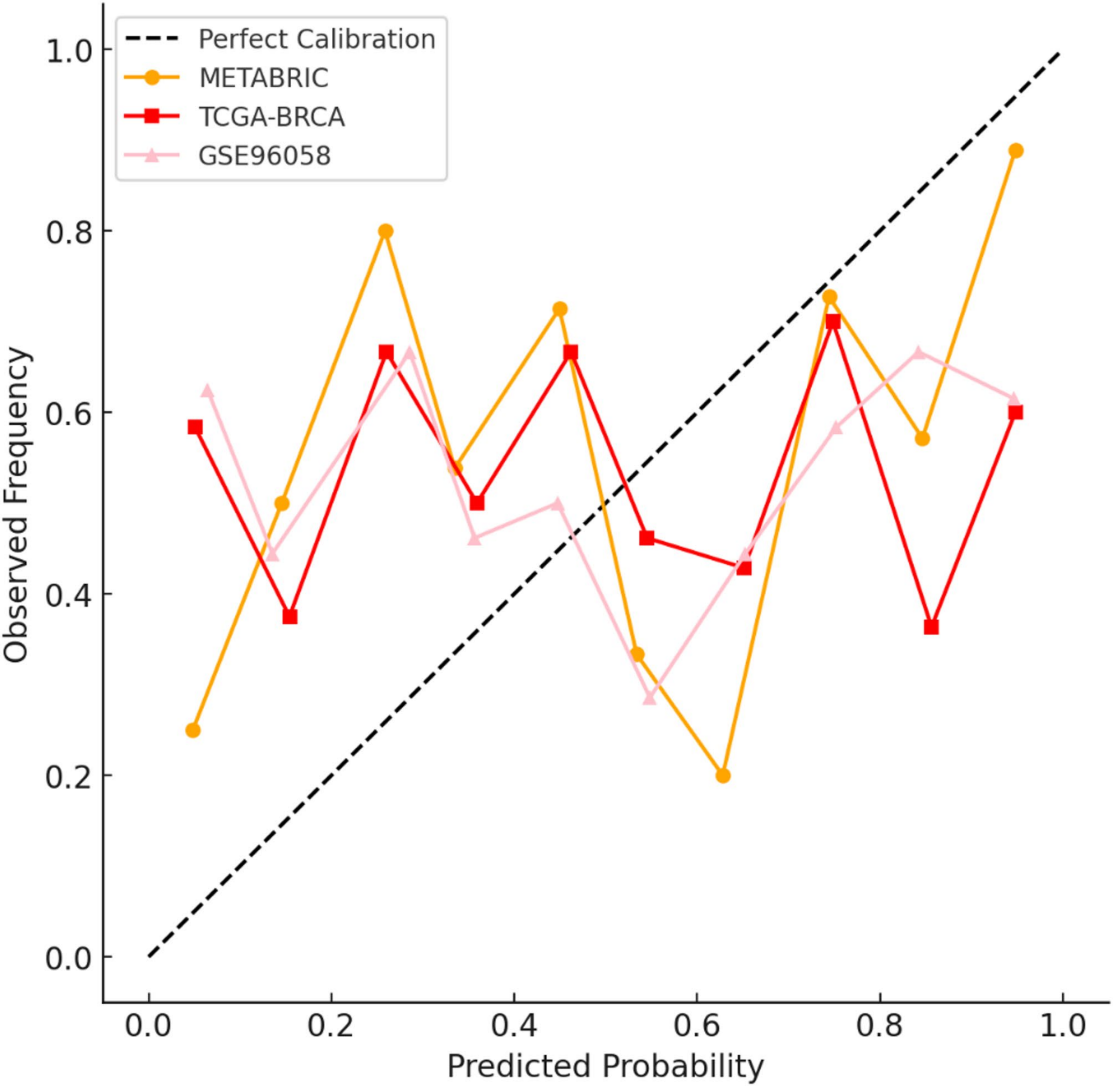
Calibration curves for each dataset.

Figure 14 presents calibration curves, revealing varying calibration performance across datasets. METABRIC’s curve closely follows the ideal calibration line, with minor overestimation of survival. TCGA-BRCA tends to underestimate survival probabilities at the lower end of the probability spectrum, with better calibration at higher probabilities. The GSE96058 dataset’s calibration curve deviates the most, indicating overconfident predictions for intermediate survival probabilities, but accurate calibration for very low and very high survival probabilities.

## Interpretations of results

### Results and their significance

By incorporating the C-index and calibration curves from the integrated approach of agent-based modelling (ABM) and gene expression profiling, we can directly observe how these methods work together. This collaboration between ABM and gene expression profiling provides valuable insights into patient outcomes and breast cancer progression. Analyzing these insights allows us to interpret the results and their potential consequences. The model’s consistent performance across METABRIC, TCGA-BRCA, and GSE96058 suggests its utility in diverse clinical settings. While batch effects were mitigated via Combat, future work should address cohort-specific biases (e.g., treatment protocols). Nevertheless, the replication of key features underscores their biological universality.

#### High C-index

ABM may provide pertinent data for risk stratification if the C-Index is near 1, leading to individuals being ranked in terms of expected survival. The model’s ranking of patients $\:\left(based\:on\:{T}_{predicted}\right)$ and their actual survival periods $\:\left({T}_{actual}\right)$ tend to be strongly linked.

#### Good calibration

If the calibration curves reveal a sufficient connection between observed and projected survival probability at various intervals, then the reliability of the model’s predictions is illustrated throughout the whole survival spectrum. This suggests that the model’s output survival probabilities—tend to be feasible to reconcile with realistic patient data and tend to be better represented by foreseeable results.

Genes that have a strong influence on predicted survival $\:\left({T}_{relapse}\right)$ when their expression levels vary $\:\left({E}_{i}\right)$ as the sensitivity analysis reveals, are inclined to have an enormous impact on the simulated tumor dynamics. Further, certain genes—known as virtual knockouts—contribute to improved survival outcomes, such as prolonged $\:\left({T}_{relapse}\right)$ and subsequent therapeutic medication generation. This emphasizes how crucial these genes are to the onset of breast cancer and implies suppressing them in real individuals may have improved therapeutic results.

To ensure the robustness of these findings, we validated the identified genes using the TCGA-BRCA and GSE96058 datasets. For example, the gene *ESR1* (estrogen receptor 1), which was identified as a key driver of tumor growth in our model, showed consistent expression patterns across all three datasets. Similarly, the gene *TP53* (tumor protein p53), associated with poor survival outcomes, was validated in both external datasets. These results confirm that the gene-expression patterns identified in our study are not dataset-specific but are biologically significant across diverse patient populations.

Gene expression data gets utilized to tailor the ABM, perhaps identifying distinct differences in tumor biology, and predicting survival results relevant to an aware patient. The model combines biological systems through updated rules, enabling us to understand the role that gene expression plays in tumor formation and survival. Contrary to certain machine learning computations, ABM provides a more palatable framework for detecting key genes and pathways.

### Key findings

Coupling gene expression profiling and agent-based modelling (ABM) into a singular, cohesive strategy led to important breakthroughs that accentuate the potential for bespoke treatment and hold wider implications for improving patient outcomes in the field of breast cancer.

- The study likely judges the integrated ABM process by using metrics like the C-Index and calibration curves. A high C-Index and good calibration mean the model makes reliable predictions of survival chances and accurately ranks patients based on predicted survival.
- The ABM study might identify important genes or pathways that influence survival outcomes through techniques like sensitivity analysis and virtual knockouts. This supports the idea that genes play a role in tumor development.
- The ABM can predict individual survival outcomes by combining patient-specific gene expression data. This suggests that the chance of survival or recurrence can be influenced, which can help with choosing treatment options.
- ABM can be used to identify genes critical for tumor growth, which can help set therapy goals and prioritize future research and drug development.
- Doctors can personalize treatment plans for each patient by using the ABM to classify patients based on the underlying tumor biology and their predicted risk.
- The ABM approach helps discover key genes or pathways, which can be used to develop targeted drugs that specifically target the biological mechanisms driving individual tumor growth.
- The ABM approach is advancing the field of precision medicine, aiming to provide cancer patients with customized and effective treatment options.

## Limitations of the study

While this study presents a novel approach to breast cancer prognosis, minimal limitations should be acknowledged. The retrospective nature of the dataset may introduce inherent biases and limit the generalizability of the findings to prospective patient populations. Additionally, the complexity of breast cancer and the multitude of factors influencing patient outcomes necessitate further exploration of additional molecular markers and clinical variables. Furthermore, external validation of the model in independent chores is crucial to assess its robustness and clinical utility.

## Conclusion

Through this investigation and the model’s functioning, the most trustworthy indicators of breast cancer patients’ prognoses emerged. The most reliable predictor was “mutation_count,” a genetic trait. Numerous studies, especially in cases of breast cancer, indicate a high correlation between genetic information and survival. Breast cancer arises from genetic mutations linked to irregularities in DNA. Learning the most frequently altered genes detected in the sample could assist in comprehending the genetic alterations that are unquestionably associated with breast cancer survival. In clinical decision-making, this might help define the significance of an improved forecast.

In the conclusion of, we used age, cell count, tumor size, lymph node involvement, and tumor grade to predict survival rates and achieved this by incorporating factors with weights determined by a mathematical technique called Singular Value Decomposition (SVD) into a logistic regression model. This model estimates the probability of survival over time. The model’s effectiveness in predicting probabilities is confirmed by its ability to achieve an AUC-ROC (Area Under the Receiver Operating Characteristic Curve) of up to 84.5% in classifying a dataset. Additionally, the model maintains a low false positive rate of around 2%, which is crucial to avoid overestimating survival rates.

Looking ahead, we are exploring the potential of combining agent-based models (ABMs) with various machine learning algorithms to leverage the strengths of both approaches for better outcomes. Additionally, high-performance computing resources can facilitate the development of more complex and data-driven ABMs. Standardizing the creation of ABMs and sharing model components could significantly accelerate progress in this field. Finally, further research using diverse datasets that consider regional tumor variations is needed to refine and improve ABMs. These advancements hold promises for ABMs to play a significant role in tailoring treatment plans for breast cancer patients.

## Data Availability

The dataset going to be used in the case study is published in Nature Communications (Pereira et al., 2016), also available in Kaggle named as Molecular Taxonomy of Breast Cancer International Consortium (METABRIC) database. The associated clinical and genomic data was downloaded from cBioPortal.In addition to that other datasets are http://cancergenome.nih.gov/ and https://www.ncbi.nlm.nih.gov/sites/GDSbrowser/.

## Abbreviations

GEP: Gene expression profiling
ABM: Agent based model
C-Index: Concordance index
AUC: Area under the receiver operating characteristics
SVD: Singular value decomposition
SV: Singular vector
ROC: Receiving operating characteristics
TPR: True positive rate
FPR: False positive rate
CDF: Cumulative distributive function
SSE: Sum of squared estimate of errors
DNA: Deoxyribonucleic acid
mRNA: Messenger ribonucleic acid
RNA-seq: Ribonucleic acid sequencing
ML: Machine learning
ANOVA: Analysis of variance
ODE: Ordinary differential equation
OS: Overall survival
DEA: Differential expression analysis
